# Supplementary material for: Ductile deformation during carbonation of serpentinized peridotite
Source: Nat Commun. 2022 Jun 16;13:3478. doi: 10.1038/s41467-022-31049-1 (PMC9203755; doi:10.1038/s41467-022-31049-1)
Supplement: Supplementary file 1 — Supplementary Information [file 41467_2022_31049_MOESM1_ESM.pdf]

Supplementary Material to: Ductile deformation during carbonation of serpentinized peridotite

Manuel D. Menzel[1,2]*, Janos L. Urai[1], Estibalitz Ukar[3], Greg Hirth[4], Alexander Schwedt[5], András Kovács[6], Lidia Kibkalo[6], Peter B. Kelemen[7]

[1] *Tectonics and Geodynamics, RWTH Aachen University, Lochnerstrasse 4-20, D-52056 Aachen, Germany*

[2] *now at: Instituto Andaluz de Ciencias de la Tierra (IACT) (CSIC-Universidad de Granada), Avenida de las Palmeras 4, 18100 Armilla, Granada, Spain*

[3] *University of Texas at Austin, Bureau of Economic Geology, TX, USA*

[4] *Brown University, Department of Earth, Environmental and Planetary Sciences, Providence, RI, USA*

[5] *RWTH Aachen University, Central Facility for Electron Microscopy, Aachen, Germany*

[6] *Ernst Ruska-Centre for Microscopy and Spectroscopy with Electrons, Forschungszentrum Jülich, Jülich, Germany*

[7] *Lamont–Doherty Earth Observatory, Columbia University, USA*

* corresponding author (manuel.menzel@csic.es)

Supplementary Notes

Supplementary Note 1: Tectonic setting of listvenite formation at Site BT1

The tectonic setting and geodynamic context of listvenite formation at Site BT1 are debated due to the lack of precise dating of carbonation. As this question is not the main topic of the manuscript but may nonetheless be of interest for many readers, we provide here a short summary and discussion of the possible tectonic settings. This discussion is based on different results and inferences from this and previous studies, in addition to thoughts and comments by the referees of this manuscript. The full review correspondence, which includes further discussion of this question, is available as a separate file.

The timing of listvenite in the Samail ophiolite is poorly constrained by one imprecise Rb-Sr isochron date of 97±29 Ma (2σ) (Falk and Kelemen, 2015; 2σ uncertainty confirmed by personal communication with L. Falk). Unpublished U/Pb formation or cooling ages (60 ± 16 and 58 ± 6 Ma) of calcite veins cutting (and thus post-dating) listvenite (Scharf et al., 2020) may indicate that listvenite formed before 60 Ma. Based on these time constraints alone, listvenite formation may have been concurrent with intra-oceanic subduction (96 to 85 Ma; e.g., Rioux et al., 2021) or continental subduction of the Arabian margin and ophiolite obduction (85 – 77 Ma; e.g., Garber et al., 2021; Warren et al., 2003). Some or all of the listvenite might also have formed during an early phase of post-obduction extension with top-to-the-NNE shear during the Maastrichtian and early Paleocene (< 74 Ma; Grobe et al., 2019; Hansmann et al., 2017; Mattern and Scharf, 2018; Scharf et al., 2021). As discussed by Kelemen et al. (2022), the 97±29 Ma isochron age (2σ; Falk & Kelemen, 2015) "yields a 93% chance (1.5σ) that the listvenites formed before 75 Ma, while subduction beneath the ophiolite was still active" (Kelemen et al., 2022). This does not exclude a post-obduction extensional setting but makes it less likely than listvenite formation during thrusting.

Early models proposed that listvenite formation may have coincided with extensional faulting, meteoric fluid circulation and elevated heat flow during the Paleogene (Nasir et al., 2007; Stanger, 1985). This model is inconsistent with the available dates, even when considering the low precision of the Rb-Sr isochron (Falk and Kelemen, 2015). The parallel alignment of listvenite bands around Site BT1 with the gently folded, low-angle contacts between the basal mantle section of the ophiolite, the metamorphic sole and the Hawasina sediments (Kelemen et al., 2022) further indicates that the listvenites formed along the low angle, basal thrust of the ophiolite, and not along steep normal faults.

Based on thermal considerations, listvenite formation during subduction infancy as recorded by the metamorphic sole (Soret et al., 2017) appears very unlikely. The metamorphic sole records substantially higher temperatures (> 400 °C and up to 900 °C; Kotowski et al., 2021; Soret et al.,

2017) than listvenites (45 – 245 °C as from clumped carbon isotopes; Beinlich et al., 2020; or 80 – 150 °C; Falk and Kelemen, 2015). However, since the contacts between listvenites and the metamorphic sole are faults with unknown amount of displacement (Kelemen et al., 2020; Menzel et al., 2020), it is unclear whether carbonation occurred in-situ, along the contact with the metamorphic sole, or if listvenite-bearing basal peridotites and the sole were tectonically juxtaposed after carbonation took place. Sr-isotope signatures rule out the metamorphic sole sampled by Hole BT1B as a CO_2-fluid source for carbonation. Instead, they point to dehydrating lower-plate (meta)sediments similar to those of the Hawasina units (de Obeso et al., 2022). This may suggest a mature subduction stage or ophiolite obduction setting, where CO_2-bearing fluids from devolatilization reactions in partially subducted sediments migrated upwards along the plate interface, forming listvenite in the colder basal mantle section of the Oman Ophiolite (de Obeso et al., 2022; Kelemen et al., 2022). Up-dip fluid migration may reconcile the conundrum that fluid quantities and CO_2-concentrations produced by dehydrating sediments are not high enough at the temperatures and the (inferred) pressure range of the BT1B listvenites to allow listvenite formation (Kelemen et al., 2022).

Listvenite formation may thus have occurred related to a mature or continental subduction / ophiolite obduction setting at around 85 – 74 Ma (e.g., Garber et al., 2021; Warren et al., 2003) in comparatively shallow (possibly 0.3 – 0.7 GPa) and cold upper-plate peridotite, due to dehydration of lower-plate sediments deeper down along the plate interface where temperatures are higher and devolatilization reactions more efficient. Such a setting is consistent with the outcrop geometry, isotope geochemistry (de Obeso et al., 2022), and the isochron age (Falk and Kelemen, 2015) of the listvenites, the requirement of a fluid source with sufficiently high CO_2 concentrations (Kelemen et al., 2022), and, broadly, with thermal gradients (Yamato et al., 2007). Therefore, based on the considerations and geochronological data outlined above, it appears most likely that listvenite formed along the thrust fault while (partial) subduction beneath the ophiolite was still active, as discussed in more detail by Kelemen et al. (2022). Still, this question remains open as the alternative of listvenite formation in an early extensional setting cannot be ruled out. Further research including detailed analysis of the structural evolution of the area and precise geochronological dating of carbonation (e.g. early magnesite veins in listvenite) could help to better constrain the geodynamic context.

Supplementary Note 2: Remark about sample provenance

All samples from BT1B have been collected from the working half of core BT1B, and sample IDs used here are a shortened version of the Oman Drilling Project standard, following the convention: “Hole”_”core”-“section”_”cm from top of section”. The archive half of core BT1B is available for viewing in the Sultanate of Oman, and abundant datasets for Hole BT1B are publically accessible

through the Supplements of the Proceedings of the Oman Drilling Project (http://publications.iodp.org/other/Oman/OmanDP.html).

Field and core samples have been collected and exported in a responsible manner and in accordance with relevant permits and local laws of the Sultanate of Oman.

Supplementary References

Beinlich, A., Plumper, O., Boter, E., Muller, I.A., Kourim, F., Ziegler, M., Harigane, Y., Lafay, R., Kelemen, P.B., Oman Drilling Project Science Team, 2020. Ultramafic Rock Carbonation: Constraints From Listvenite Core BT1B, Oman Drilling Project. J Geophys Res-Sol Ea 125, e2019JB019060.

de Obeso, J.C., Kelemen, P.B., Leong, J.M., Menzel, M.D., Manning, C.E., Godard, M., Cai, Y., Bolge, L., Oman Drilling Project Phase 1 Science, P., 2022. Deep Sourced Fluids for Peridotite Carbonation in the Shallow Mantle Wedge of a Fossil Subduction Zone: Sr and C Isotope Profiles of OmanDP Hole BT1B. Journal of Geophysical Research: Solid Earth 127, e2021JB022704.

Falk, E.S., Kelemen, P.B., 2015. Geochemistry and petrology of listvenite in the Samail ophiolite, Sultanate of Oman: Complete carbonation of peridotite during ophiolite emplacement. Geochimica et Cosmochimica Acta 160, 70-90.

Garber, J.M., Rioux, M., Searle, M.P., Kylander-Clark, A.R.C., Hacker, B.R., Vervoort, J.D., Warren, C.J., Smye, A.J., 2021. Dating Continental Subduction Beneath the Samail Ophiolite: Garnet, Zircon, and Rutile Petrochronology of the As Sifah Eclogites, NE Oman. Journal of Geophysical Research: Solid Earth 126, e2021JB022715.

Grobe, A., Virgo, S., von Hagke, C., Urai, J.L., Littke, R., 2018. Multiphase Structural Evolution of a Continental Margin During Obduction Orogeny: Insights From the Jebel Akhdar Dome, Oman Mountains. Tectonics 37, 888-913.

Grobe, A., von Hagke, C., Littke, R., Dunkl, I., Wübbeler, F., Muchez, P., Urai, J.L., 2019. Tectono-thermal evolution of Oman's Mesozoic passive continental margin under the obducting Semail Ophiolite: a case study of Jebel Akhdar, Oman. Solid Earth 10, 149-175.

Hansman, R.J., Ring, U., Thomson, S.N., den Brok, B., Stübner, K., 2017. Late Eocene Uplift of the Al Hajar Mountains, Oman, Supported by Stratigraphy and Low-Temperature Thermochronology. Tectonics 36, 3081-3109.

Kelemen, P.B., Carlos de Obeso, J., Leong, J.A., Godard, M., Okazaki, K., Kotowski, A.J., Manning, C.E., Ellison, E.T., Menzel, M.D., Urai, J.L., Hirth, G., Rioux, M., Stockli, D.F., Lafay, R., Beinlich, A.M., Coggon, J.A., Warsi, N.H., Matter, J.M., Teagle, D.A.H., Harris, M., Michibayashi, K., Takazawa, E., Al Sulaimani, Z., the Oman Drilling Project Science Team, 2022. Listvenite Formation During Mass Transfer into the Leading Edge of the Mantle Wedge: Initial Results from Oman Drilling Project Hole BT1B. Journal of Geophysical Research: Solid Earth 127, e2021JB022352.

Kelemen, P.B., Matter, J.M., Teagle, D.A.H., Coggon, J.A., and the Oman Drilling Project Science Team, 2020. Site BT1: fluid and mass exchange on a subduction zone plate boundary, in: Kelemen, P.B., Matter, J.M., Teagle, D.A.H., Coggon, J.A., et al. (Eds.), Proceedings of the Oman Drilling Project. International Ocean Discovery Program, College Station, TX.

Kotowski, A.J., Cloos, M., Stockli, D.F., Bos Orent, E., 2021. Structural and Thermal Evolution of an Infant Subduction Shear Zone: Insights From Sub-Ophiolite Metamorphic Rocks Recovered From Oman Drilling Project Site BT-1B. Journal of Geophysical Research: Solid Earth 126, e2021JB021702.

Mattern, F., Scharf, A., 2018. Postobductional extension along and within the Frontal Range of the Eastern Oman Mountains. Journal of Asian Earth Sciences 154, 369-385.

Menzel, M.D., Urai, J.L., de Obeso, J.C., Kotowski, A., Manning, C.E., Kelemen, P.B., Kettermann, M., Jesus, A.P., Harigane, Y., the Oman Drilling Project Phase 1 Science, T., 2020. Brittle Deformation of Carbonated Peridotite—Insights From Listvenites of the Samail Ophiolite (Oman Drilling Project Hole BT1B). Journal of Geophysical Research: Solid Earth 125, e2020JB020199.

Nasir, S., Al Sayigh, A.R., Al Harthy, A., Al-Khirbash, S., Al-Jaaidi, O., Musllam, A., Al-Mishwat, A., Al-Bu'saidi, S., 2007. Mineralogical and geochemical characterization of listwaenite from the Semail Ophiolite, Oman. Geochemistry 67, 213-228.

Rioux, M., Garber, J.M., Searle, M., Kelemen, P., Miyashita, S., Adachi, Y., Bowring, S., 2021. High-Precision U-Pb Zircon Dating of Late Magmatism in the Samail Ophiolite: A Record of Subduction Initiation. Journal of Geophysical Research: Solid Earth 126, e2020JB020758.

Scharf, A., Mattern, F., Al-Wardi, M., Frijia, G., Moraetis, D., Pracejus, B., Bauer, W., Callegari, I., 2021. Chapter 2 Tectonostratigraphy of the eastern part of the Oman Mountains, in: Scharf, A., Mattern, F., Al-Wardi, M., Frijia, G., Moraetis, D., Pracejus, B., Bauer, W., Callegari, I. (Eds.), The Geology and Tectonics of the Jabal Akhdar and Saih Hatat Domes, Oman Mountains. Geological Society of London, p. 0.

Scharf, A., Mattern, F., Bolhar, R., Bailey, C. M., & Ring, U., 2020. U-Pb dating of postobductional carbonate veins in listwaenite of the Oman Mountains near Fanja. Presented at Proceedings of the International Conference on Ophiolites and the Oceanic Lithosphere: Results of the Oman Drilling Project and Related Research, Sultan Qaboos University, Oman.

Soret, M., Agard, P., Dubacq, B., Plunder, A., Yamato, P., 2017. Petrological evidence for stepwise accretion of metamorphic soles during subduction infancy (Semail ophiolite, Oman and UAE). Journal of Metamorphic Geology 35, 1051-1080.

Stanger, G., 1985. Silicified serpentinite in the Semail nappe of Oman. Lithos 18, 13-22.

Warren, C.J., Parrish, R.R., Searle, M.P., Waters, D.J., 2003. Dating the subduction of the Arabian continental margin beneath the Semail ophiolite, Oman. Geology 31, 889-892.

Yamato, P., Agard, P., GoffÉ, B., De Andrade, V., Vidal, O., Jolivet, L., 2007. New, high-precision P–T estimates for Oman blueschists: implications for obduction, nappe stacking and exhumation processes. Journal of Metamorphic Geology 25, 657-682.

Supplementary Tables

Supplementary Table S1a: Overview of studied thin sections of samples consisting of foliated serpentinites (highlighted green) and listvenites with ductile deformation structures in Oman Drilling Project Hole BT1B (lat: 23.3643° / lon: 58.1825°). SPO = shape preferred orientation; CPO = crystallographic preferred orientation. Depth is down-hole distance relative to the top of Hole BT1B.

Sample ID (exp. 5057_4B)	Lithology comment	Depth (m)	ViP	SEM	EBSD
BT1B_14-3_60-66	Foliated listvenite with SPO magnesite and weak CPO of quartz, folded early Fe-magnesite/Fe-oxide veins, Cr-Spinel porphyroclasts with strain shadows	25.80	x	x	x
BT1B_14-3_77-80	Foliated listvenite with SPO and CPO of magnesite spheroids (and locally dendrites), transposition of early Fe-magnesite veins, boudinage of Cr-spinel porphyroclasts, spinel σ-clasts	25.96	x	x	x
BT1B_15-1_32-35	Foliated listvenite with SPO of magnesite spheroids (and locally dendrites), folded Fe-magnesite/Fe-oxide veins, Cr-Spinel porphyroclasts with strain shadows	26.27	x	x	
BT1B_16-3_13-16	Massive listvenite with locally folded and transposed early magnesite veins	28.93			
BT1B_16-3_28-31	Foliated listvenite with high vein density, locally folded veins, (dendritic) magnesite SPO, quartz CPO	29.09	x	x	x
BT1B_20-1_15-18	Foliated listvenite with mylonitic appearance, similar to BT1B_20-1_64-68	39.35			
BT1B_20-1_64-68	Foliated listvenite with mylonitic appearance due to strong SPO of magnesite spheroids / dendrites; magnesite CPO, transposed early magnesite veins, strain shadows on Cr-spinel porphyroclasts, aligned Fe-oxide aggregates	39.84	x	x	x
BT1B_21-3_35-40	Foliated listvenite, variably sheared on thin section scale. Folded early magnesite veins; SPO and CPO of magnesite dendrites.	44.39	x	x	x
BT1B_26-3_28-31	Foliated listvenite with SPO of magnesite, folded early magnesite veins, Cr-Spinel porphyroclasts with strain shadows	56.47		x	
BT1B_26-3_53-58	Listvenite, weak foliated (grey-red); localized shear zone (c. 0.7 cm thick), veins	56.72			
BT1B_27-2_6-8	Massive listvenite with local shear zone consisting of strongly folded and transposed magnesite veins	58.36	x	x	
BT1B_31-4_12-14	Strongly veined, foliated listvenite, with local CPO of quartz in between branched, subparallel magnesite-dolomite veins that form an s-c resembling fabric; aligned Fe-oxide aggregates	66.08	x	x	x
BT1B_32-1_5-9	Strongly veined, foliated fine-grained listvenite; branched, thin subparallel magnesite veins; Cr-spinel σ-clasts; boudinage of fine grained magnesite-Fe-oxide aggregates	66.70	x	x	
BT1B_32-1_17-19	Strongly veined, foliated listvenite; like BT1B_32-1_5-9	66.82			x
BT1B_39-2_67-72	Mesh/bastite serpentinite with carbonate veins, locally sheared serpentine with SPO subparallel to veins; folded serpentine-oxide veins; local cataclasite cut by faults, serpentine and carbonate veins	83.45	x	x	
BT1B_39-4_14-18	Foliated serpentinite with branched carbonate veins forming an s-c resembling fabric. Aligned Fe-oxide aggregates; SPO and CPO of serpentine, locally folded and cut by cataclasite	84.36	x	x	
BT1B_42-2_19-24	Sheared and folded serpentinite with local replacement by dolomite and quartz veins along the (folded) serpentine cleavage	91.75	x	x	
BT1B_44-4_78-81	Quartz-rich, talc-bearing transition between serpentinite and listvenite, local quartz CPO and talc SPO, transposed early veins	100.20	x	x	
BT1B_47-3_15-19	Listvenite with spheroidal-euhedral magnesite, locally folded Fe-magnesite veins	107.83			
BT1B_51-1_20-25	Massive listvenite cut by a veined shear zone; local CPO of quartz, minor assymetric folding of magnesite veins in the sheared part	115.65	x	x	
BT1B_67-2_36-40	Massive listvenite cut by a veined shear zone	162.11	x	x	x

BT1B_74-1_59-61	Mesh/bastite serpentinite and mylonitic serpentinite. Mylonitic serpentinite: serpentine SPO and CPO, kinking, grain size reduction	182.94	x	x	x
BT1B_78-2_34-38	Foliated, dolomite-rich listvenite; strong CPO of dolomite, with SPO of elongated carbonate grains and aligned vein fragments	195.87	x	x	x

Supplementary Table S1b: Field samples of foliated serpentinites with ductile deformation structures.

Sample ID	Lithology comment	latitude / longitude	ViP	SEM	EBSD
OM20-12	Foliated, recrystallized serpentinite with flattened magnetite mesh cells, interlocked antigorite, and deformation lamellae and kinking of lizardite	23.3945 / 58.1803	x		x
OM20-13	Foliated serpentinite with optical CPO of lizardite matrix and parallel chrysotile veins; flattened magnetite mesh cells	23.3981 / 58.1800	x		

Supplementary Figures

Fig. S1 Field photographs of foliated listvenites on polished Wadi walls (lat. 23.3580°/ lon. 58.2050°). (a) white carbonate veins crosscutting the foliation; (b) localized shear zone in listvenite; (c) parallel red Fe-oxide bearing veins in matrix listvenite, cut by carbonate-quartz veins that are offset by faults. Coin for scale is 1.7 cm.

Fig. S2 Examples of archive-half split core images of sheared serpentinites in Hole BT1B. Core images are oriented upright, with the z direction in core reference frame (CRF) towards the bottom and x out of plane. All split core images of Hole BT1B are publically available as supplements of the Oman Drilling project (http://publications.iodp.org/other/Oman/OmanDP.html). The core diameter is 6.3 cm in a & b, and 4.7 cm in c –e.

Fig. S3 Examples of archive-half split core images of deformed listvenite from Hole BT1B. (a) ellipsoidal foliated listvenite with magnesite (Mgs) shape preferred orientation (sub-parallel to red oxide veins, not visible macroscopically); (b) grey bands of listvenite mylonite in foliated listvenite; (c) foliated and folded listvenite; (d) vein-foliated, hematite-rich listvenite; (e) foliated dolomite-rich listvenite close to the basal fault; the foliation is cut by cataclasite in the upper part. Similar listvenites are observed in polished Wadi walls in the field (Fig. S1). Core diameter is 6.3 cm in a –d, and 4.7 cm in e.

Fig. S4 Cr-Spinel microstructures. (a) primary magmatic microstructure of Cr-spinel (Cr-Sp), in mesh/bastite serpentinite. (b) Serpentine (Serp) foliation bending around Cr-spinel. (c) Fragmented Cr-spinel with carbonate (Carb) precipitated in the interstices in carbonate-bearing serpentinite. (d) Cr-spinel with hematite (Hem) strain shadow forming a sigma-clast, in strongly veined listvenite. (a & b: combined reflected and transmitted crossed-polarized (xpol) light with 1λ-plate; c & d: reflected light).

Fig. S5 Asymmetric folding in carbonate-bearing serpentinites. (a) Folded serpentine aggregate with interstitial carbonate and minor quartz (xpol with 1λ-plate). (b) Folded serpentine-Fe-oxide vein, cut and transposed by faults and quartz veins (ppol).

Fig. S6 Shape preferred orientation (SPO) of magnesite and porosity in foliated listvenite from µ-ct (BT1B_14-3_60-66). (a) selected subvolume of the measured sample, rendering of original data with enhanced contrast. White areas are Fe-rich magnesite or Fe-oxides, black dots are pores. (b) Segmented magnesite particles, view onto the yz-plane (directions in core reference frame, CRF). (c) Segmented magnesite particles, view onto the foliation plane (xz-plane). In the segmented images, pores and Fe-oxide inclusions where included in the magnesite particles as they are mostly hosted therein; the transparent matrix is quartz. (d –f): Volume rendering of segmented porosity for the same view directions as in a) to c). The long edge of the prism in (a & d) and of the slices/projections in (b, c, e, f) is 645 µm.

Fig. S7 Foliated listvenite BT1B_14-3_60-66. (a) Composite-color EDS map of folded Fe-magnesite/hematite veins in foliated listvenite, with Cr-spinel porphyroclasts. Late dolomite veins cut the foliation; (b) aligned magnesite ellipsoids in quartz matrix (xpol with 1λ-plate); (c) EBSD phase map of an area where magnesite has an SPO, with the inset showing the location of Fig. 6a (see also Fig. S20).

Fig. S8 Shape and crystal preferred orientations in foliated listvenite (BT1B_14-3_60-66). (a) Magnesite orientation map (inverse polefigure colorscale see inset; showing each grain plotted in x direction). (b) Quartz orientation map (colorscale plotted in y direction). (c) Histogram of the grain elongation angle relative to the image horizontal in thin section, for magnesite with aspect ratio > 1.5. (e) Pole figures of the orientation distribution function for the c-, a-and m-axes of magnesite (n = 1351); polefigures of c-, a-and m-axes of quartz (n = 6754). Polefigures are lower hemisphere and one point per grain; the contour color scale is in multiples of a random distribution. The sample orientation is YEBSD= up-Hole (-z) in core reference frame (CRF), x(CRF) is into the plane.

Fig. S9 Foliated listvenite with transposed early magnesite veins (BT1B_14-3_77-80). (a) Thin section overview (ViP ppol); (b) composite-color EDS map showing early, locally transposed Fe-magnesite (Fe-Mgs) veins, cut by a quartz-magnesite and late dolomite vein. (c, d) detail of vein transposition, with consecutive magnesite generations overgrowing the transposed Fe-magnesite vein core (ViP ppol, and SEM-CL).

Fig. S10 Shape and crystal preferred orientation of magnesite in foliated ellipsoidal listvenite (BT1B_14-3_77-80). (a) Aligned magnesite ellipsoids in quartz matrix; (b) optical CL image segmented for quartz (left) and magnesite (right), with grain boundaries from EBSD; magnesite has violet to pink luminescent, quartz shows two distinct luminescence colors. Single grains often contain both types of luminescence. (c) Magnesite orientation map, with colorscale based on the inverse polefigure (inset; showing each grain plotted in x direction and with point size relative to grain size); the relation to the core reference frame is given in the upper right; (d) Quartz orientation map and inverse polefigure; e) Pole figures of the orientation distribution function for the c-, e-, r-, f-, a-and m-axes of magnesite (n = 3205); polefigures of c-, a-and m-

axes of quartz (n = 3060). Polefigures are lower hemisphere and one point per grain; the contour color scale is in multiples of a random distribution.

Fig. S11 Misorientation to mean grain orientation maps for (a) magnesite and (b) quartz (BT1B_14-3_77-80; c.f. Fig. S10). (c, e) Misorientation-angle distribution between neighboring (sub)grains (=correlated), related to every other grain (uncorrelated) and random (uniform) (grain segmentation angle 2°). (d, f) Histograms of the grain elongation angle relative to the image horizontal in thin section, for magnesite and quartz grains with aspect ratio > 1.5.

Fig. S12 Folded early magnesite veins and fold axial cleavage in the matrix (BT1B_16-3_28-31). (a) crossed polarized micrograph with 1λ-plate; measured EBSD areas are marked; (b) Quartz orientation map and pole figures of quartz in EBSD area 2; (c) Polefigures of magnesite and (d) quartz in area 2, showing a CPO of quartz with [0001] parallel to the fold axial cleavage.

Fig. S13 Shape and crystal preferred orientations in listvenite with mylonitic appearance (BT1B_20-1_64-68). (a) Thin section partial scan overview (image width 2.5 cm). (b) Magnesite orientation map colored according to the trigonal inverse pole figure color scale (see inset in upper right), in the area indicated in a). (c) Rose diagram of the orientation of the long axis of non-circular hematite grains in thin-section surface (from image analysis of a ViP reflected light scan), superposed on the magnesite c-axis pole figure from the EBSD map in b), excluding the vein (1 point per grain). (d) Large area BSE panorama, showing fragmented Cr-spinel in a foliated listvenite matrix with aligned hematite aggregates. Images and rose diagram are rotated into the EBSD reference frame for comparison.

Fig. S14 Shape and crystal preferred orientation of magnesite in listvenite with mylonitic appearance (small area in the marked field in Fig. S13a; sample BT1B_20-1_64-68). (a) BSE image showing bimodal grain size distribution of magnesite ellipsoids vs. aligned dendrites in the matrix. (b) Pole figure of coarse grained magnesite (n = 3254); (c) Pole figure of dendritic magnesite with equivalent radius (EqR) < 7 µm (n = 1338), (lower hemisphere, 1PPG). (d) magnesite orientation map colored according to the trigonal inverse pole figure color scale (x to E); fine grained magnesite is plotted without grain boundaries. (e) Kernel average misorientation thresholded to 3°.

Fig. S15 Thin section overview of sample BT1B_21-3_35-40. (a) ViP ppol (CRF: core reference frame orientation); (b) composite EDS phase map. (c & d) Detail of folded magnesite vein with dendritic overgrowth (xpol with 1λ-plate; BSE).

Fig. S16 Folded early magnesite veins and fold axial cleavage in the matrix (BT1B_21-3_35-40). (a) plain polarized micrograph; (b, c) Quartz orientation map and pole figures of quartz (all points) in the area corresponding to the red frame, showing a weak local cpo of quartz with [0001] parallel to the fold axial cleavage.

Fig. S17 SPO and CPO in listvenite with strongly aligned magnesite dendrites (BT1B_21-3_35-40). (a) Magnesite orientation map colored according to the trigonal inverse pole figure color scale (inset, x to E); (b) Pole figures of magnesite (n = 3054); (c) EBSD phase map; (d) Pole figures of quartz (n = 2049). Pole

figures are for the central part of the area only, excluding the magnesite veins at the top and bottom. Polefigures are lower hemisphere and one point per grain; the contour color scale is in multiples of a random distribution.

Fig. S18 Strong CPO in foliated dolomite-rich listvenite close to the basal fault (BT1B_78-2_34-38). (a, b) ViP xpol images at 0 and 45°polarization angles, showing a cpo. (c) EBSD phase map; (d) dolomite orientation map (ipf colorscale to x; see inverse pole figures for colorscale); (e) 1-point-per-grain pole figure of orientation distribution function and inverse pole figures, showing a strong preferred orientation of dolomite with [0001] perpendicular to the foliation, and an a-/ m-axes girdle distribution in the foliation plane. The EBSD x-axis corresponds here to the downdip direction of Hole BT1B (≈ 75°inclined).

Fig. S19 Location, FIB preparation and TEM images of ellipsoidal magnesite in foliated listvenite. (a) EBSD magnesite orientation map (orientation colorscale in z-direction with z out of plane); the FIB section was prepared parallel to the magnesite elongation direction and cutting several low-angle boundaries (black circle and yellow dotted line; c.f. Fig. 6 a). (b) FIP milling; (c) SEM image of FIB lamella attached to grid. (d) Bright-field image of the FIB section; (b) HAADF overview image of the area containing the sealed nano-fracture shown in Fig. 6 b. The sealed nano-fracture is oriented at high angle to the overall elongation direction of the magnesite grain.

Fig. S20 Relative age relations of carbonation reaction and deformation structures in serpentinites and listvenites of core BT1B. Cross-correlation between events in serpentinite relative to those in listvenite is uncertain.

Supplementary figures

Ductile deformation during carbonation of serpentinized peridotite

Manuel D. Menzel[1,2]*, Janos L. Urai[1], Estibalitz Ukar[3], Greg Hirth[4], Alexander Schwedt[5], András Kovács[6], Lidia Kibkalo[6], Peter B. Kelemen[7]

1 Tectonics and Geodynamics, RWTH Aachen University, Lochnerstrasse 4-20, D-52056 Aachen, Germany
2 now at: Instituto Andaluz de Ciencias de la Tierra (IACT) (CSIC-Universidad de Granada), Avenida de las Palmeras 4, 18100 Armilla, Granada, Spain
3 University of Texas at Austin, Bureau of Economic Geology, TX, USA
4 Brown University, Department of Earth, Environmental and Planetary Sciences, Providence, RI, USA
5 RWTH Aachen University, Central Facility for Electron Microscopy, Aachen, Germany
6 Ernst Ruska-Centre for Microscopy and Spectroscopy with Electrons, Forschungszentrum Jülich Jülich, Germany
7 Lamont–Doherty Earth Observatory, Columbia University, USA

* corresponding author (manuel.menzel@emr.rwth-aachen.de)

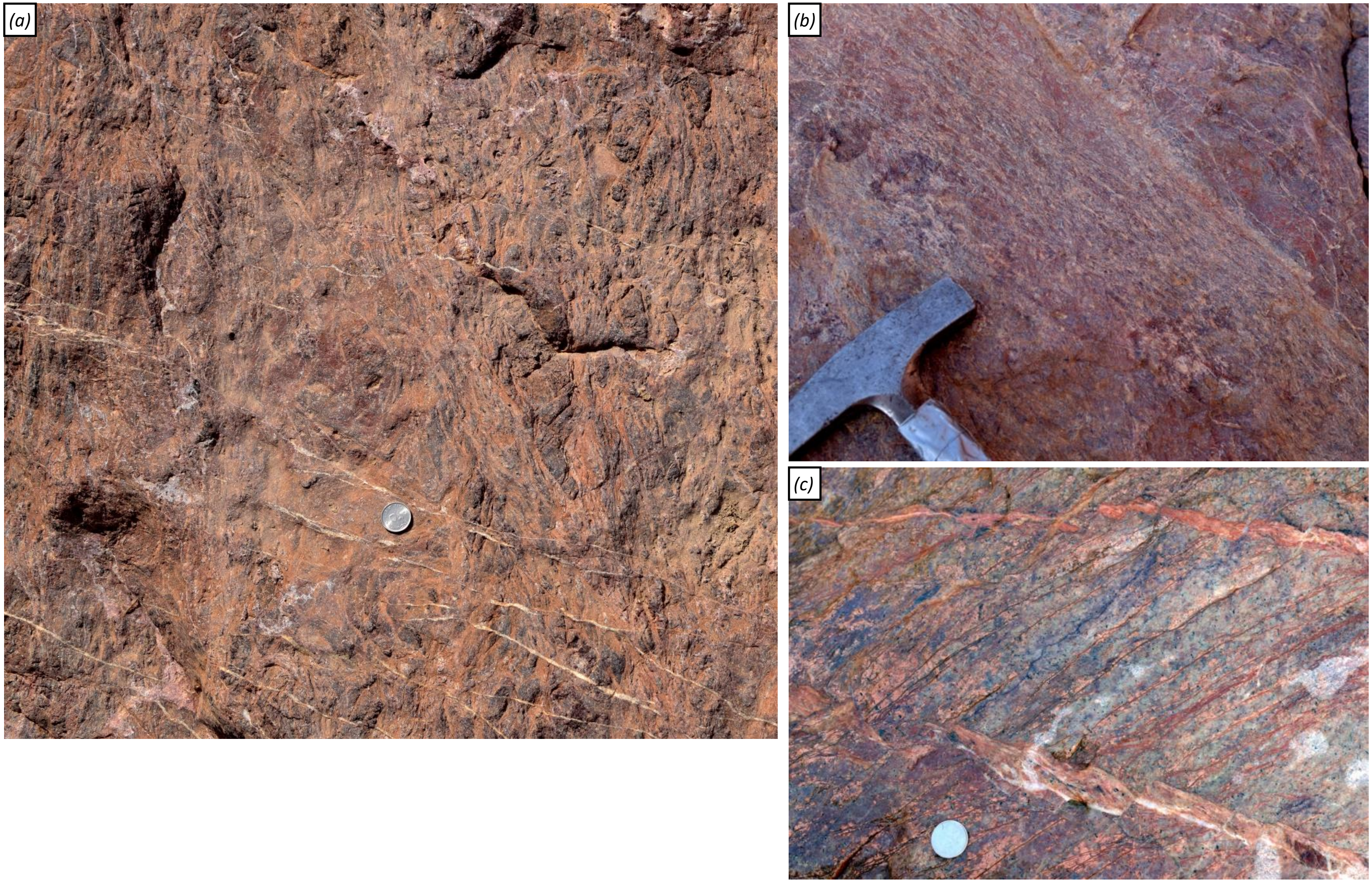

***Fig. S1** Field photographs of foliated listvenites on polished Wadi walls (lat. 23.3580° / lon. 58.2050°). (a) white carbonate veins crosscutting the foliation; (b) localized shear zone in listvenite; (c) parallel red Fe-oxide bearing veins in matrix listvenite, cut by carbonate-quartz veins that are offset by faults. Coin for scale is 1.7 cm.*

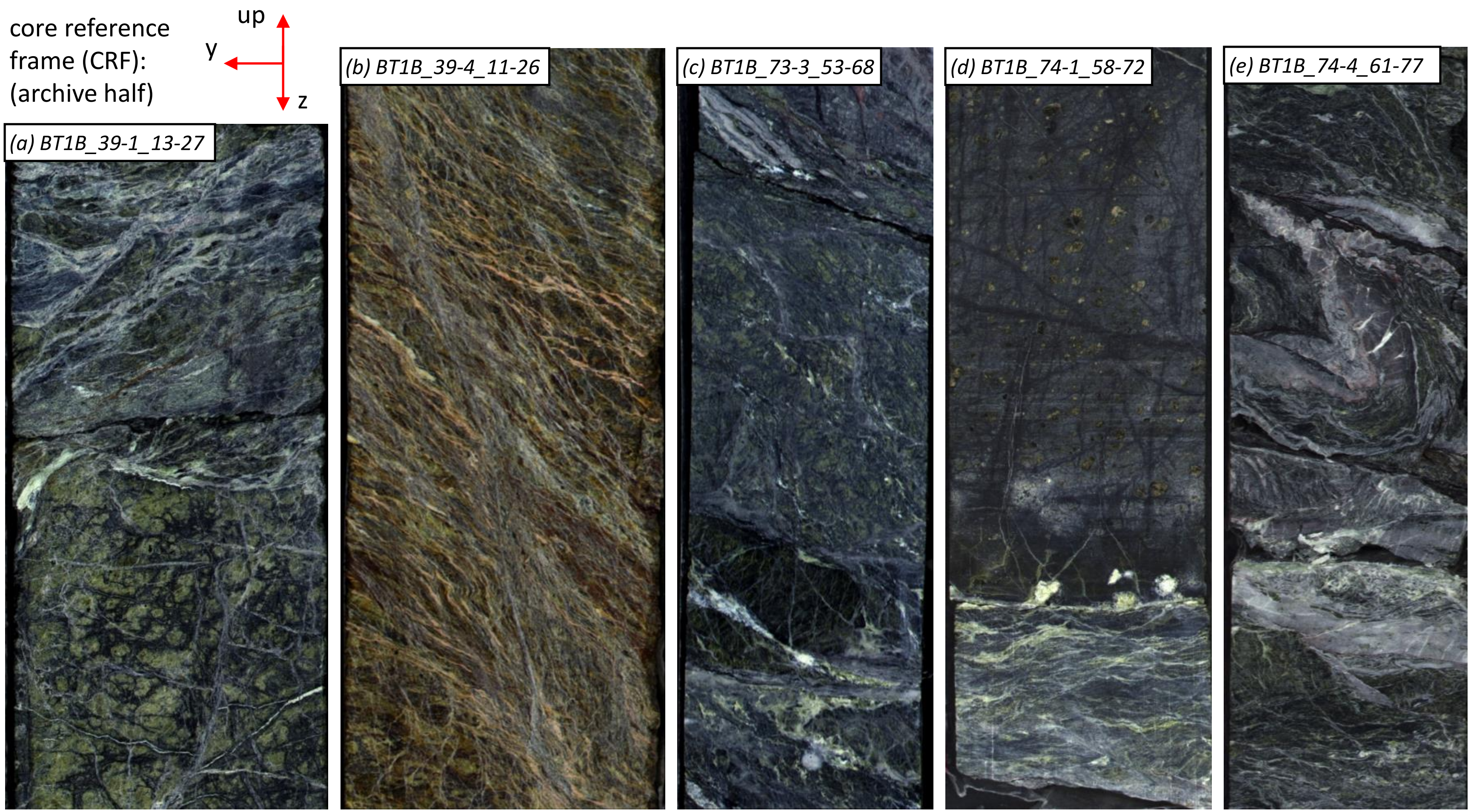

Fig. S2 *Examples of archive-half split core images of sheared serpentinites in Hole BT1B. Core images are oriented upright, with the z direction in core reference frame (CRF) towards the bottom and x out of plane. All split core images of Hole BT1B are publically available as supplements of the Oman Drilling project (http://publications.iodp.org/other/Oman/OmanDP.html). The core diameter is 6.3 cm in a & b, and 4.7 cm in c – e.*

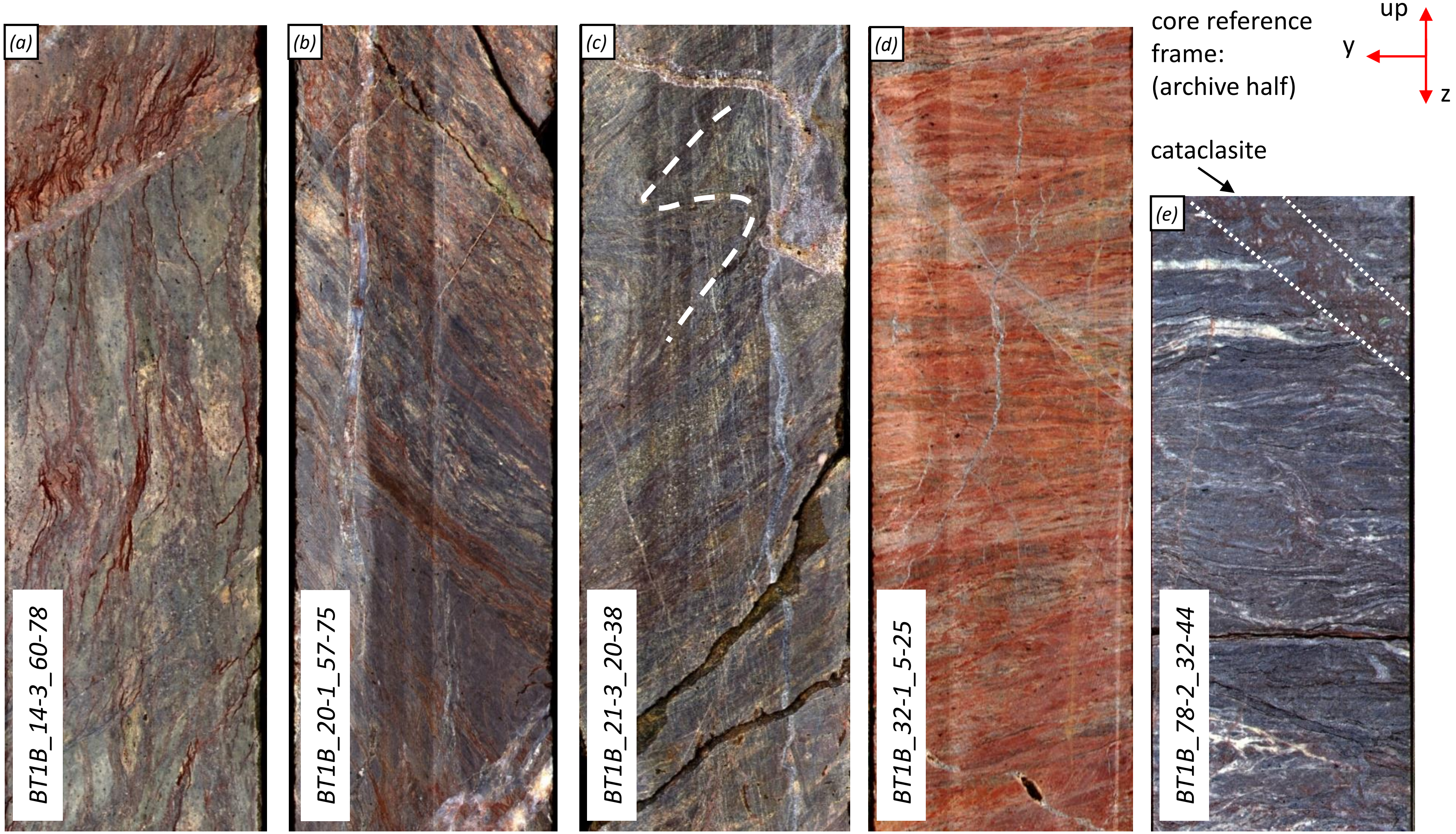

Fig. S3 *Examples of archive-half split core images of deformed listvenite from Hole BT1B. (a) ellipsoidal foliated listvenite with magnesite (Mgs) shape preferred orientation (sub-parallel to red oxide veins, not visible macroscopically); (b) grey bands of listvenite mylonite in foliated listvenite; (c) foliated and folded listvenite; (d) vein-foliated, hematite-rich listvenite; (e) foliated dolomite-rich listvenite close to the basal fault; the foliation is cut by cataclasite in the upper part. Similar listvenites are observed in polished Wadi walls in the field (Fig. S1). Core diameter is 6.3 cm in a – d, and 4.7 cm in e.*

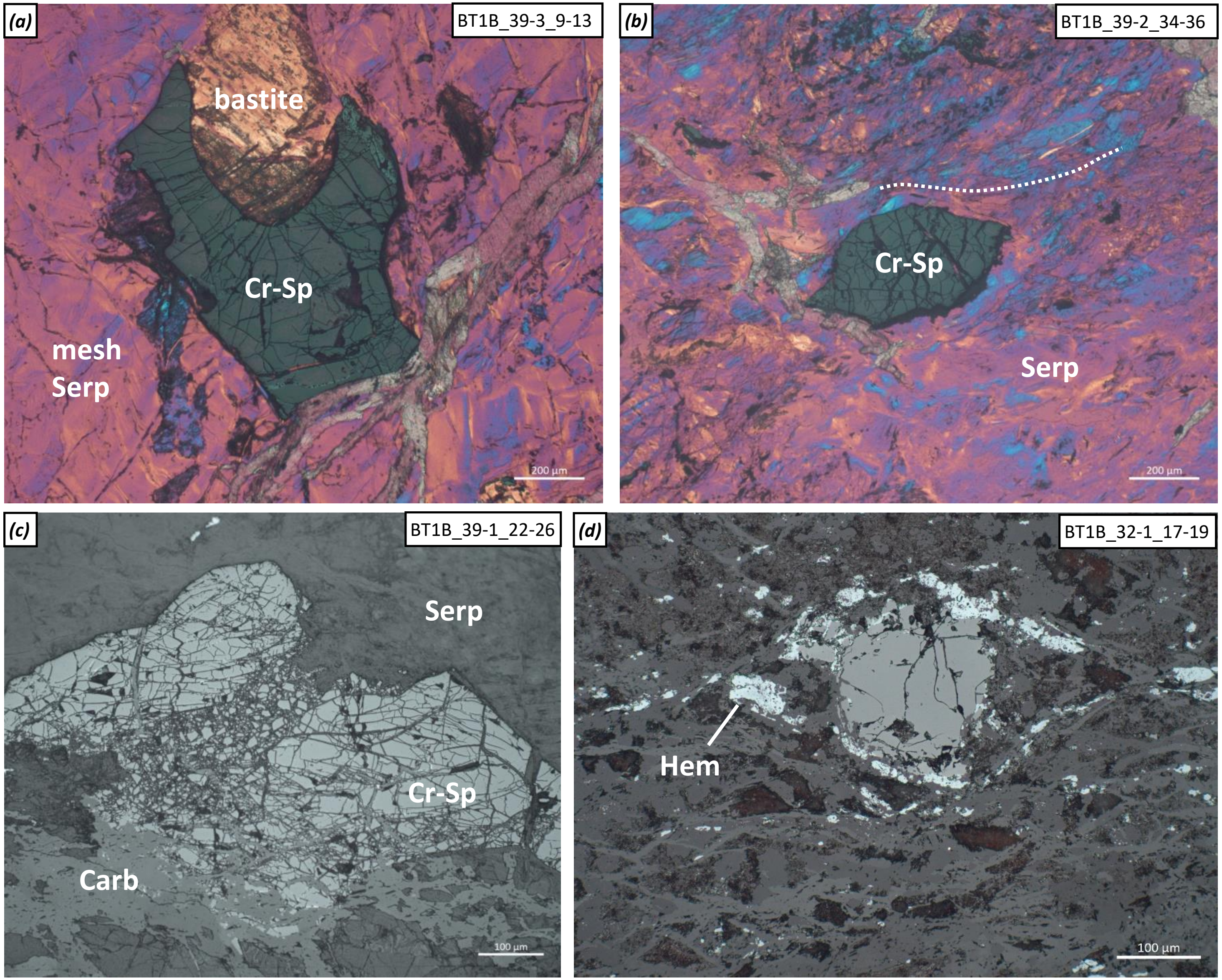

Fig. S4 Cr-Spinel microstructures

(a) primary magmatic microstructure of Cr-spinel (Cr-Sp), in mesh/bastite serpentinite. (b) Serpentine (Serp) foliation bending around Cr-spinel. (c) Fragmented Cr-spinel with carbonate (Carb) precipitated in the interstices in carbonate-bearing serpentinite. (d) Cr-spinel with hematite (Hem) strain shadow forming a sigma-clast, in strongly veined listvenite.

(a & b: combined reflected and transmitted crossed-polarized (xpol) light with 1λ-plate; c & d: reflected light).

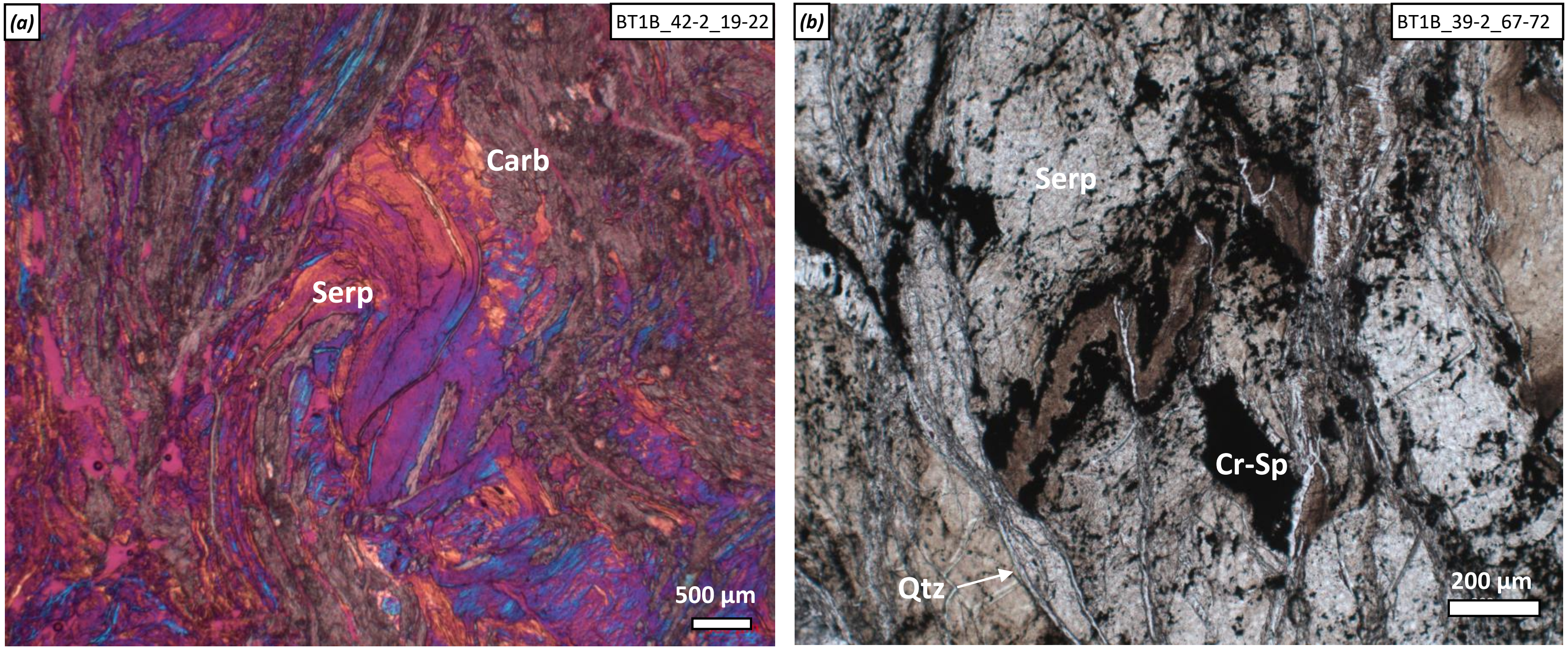

Fig. S5 Asymmetric folding in carbonate-bearing serpentinites

(a) Folded serpentine aggregate with interstitial carbonate and minor quartz (xpol with 1λ-plate). (b) Folded serpentine-Fe-oxide vein, cut and transposed by faults and quartz veins (ppol).

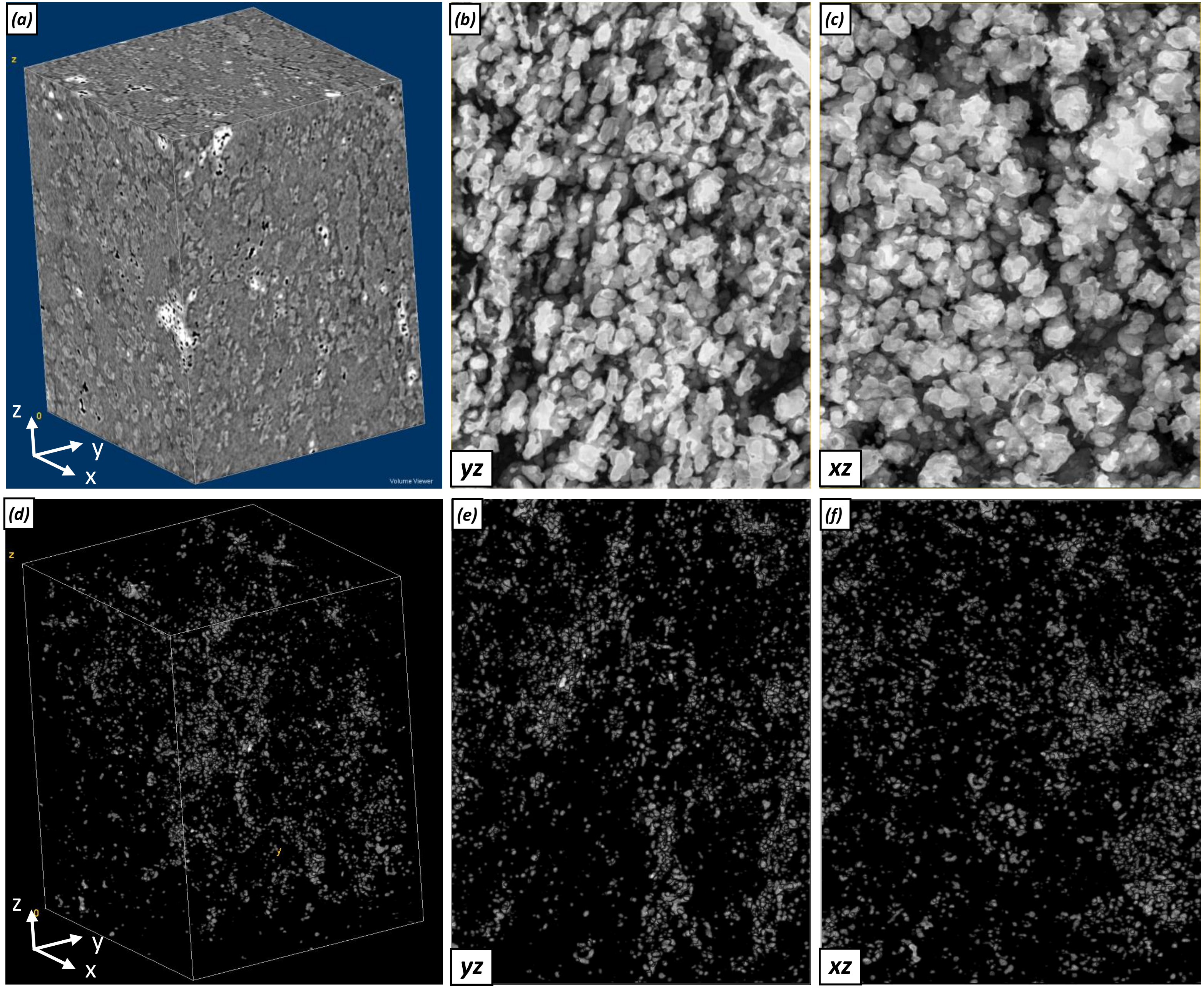

Fig. S6 *SPO of magnesite and porosity in foliated listvenite from μ-ct (BT1B_14-3_60-66). (a) selected subvolume of the measured sample, rendering of original data with enhanced contrast. White areas are Fe-rich magnesite or Fe-oxides, black dots are pores. (b) Segmented magnesite particles, view onto the yz-plane (directions in CRF). (c) Segmented magnesite particles, view onto the foliation plane (xz-plane). In the segmented images, pores and Fe-oxide inclusions where included in the magnesite particles as they are mostly hosted therein; the transparent matrix is quartz. (d – f): Volume rendering of segmented porosity for the same view directions as in a) to c). The long edge of the prism in (a & d) and of the slices/projections in (b, c, e, f) is 645 μm.*

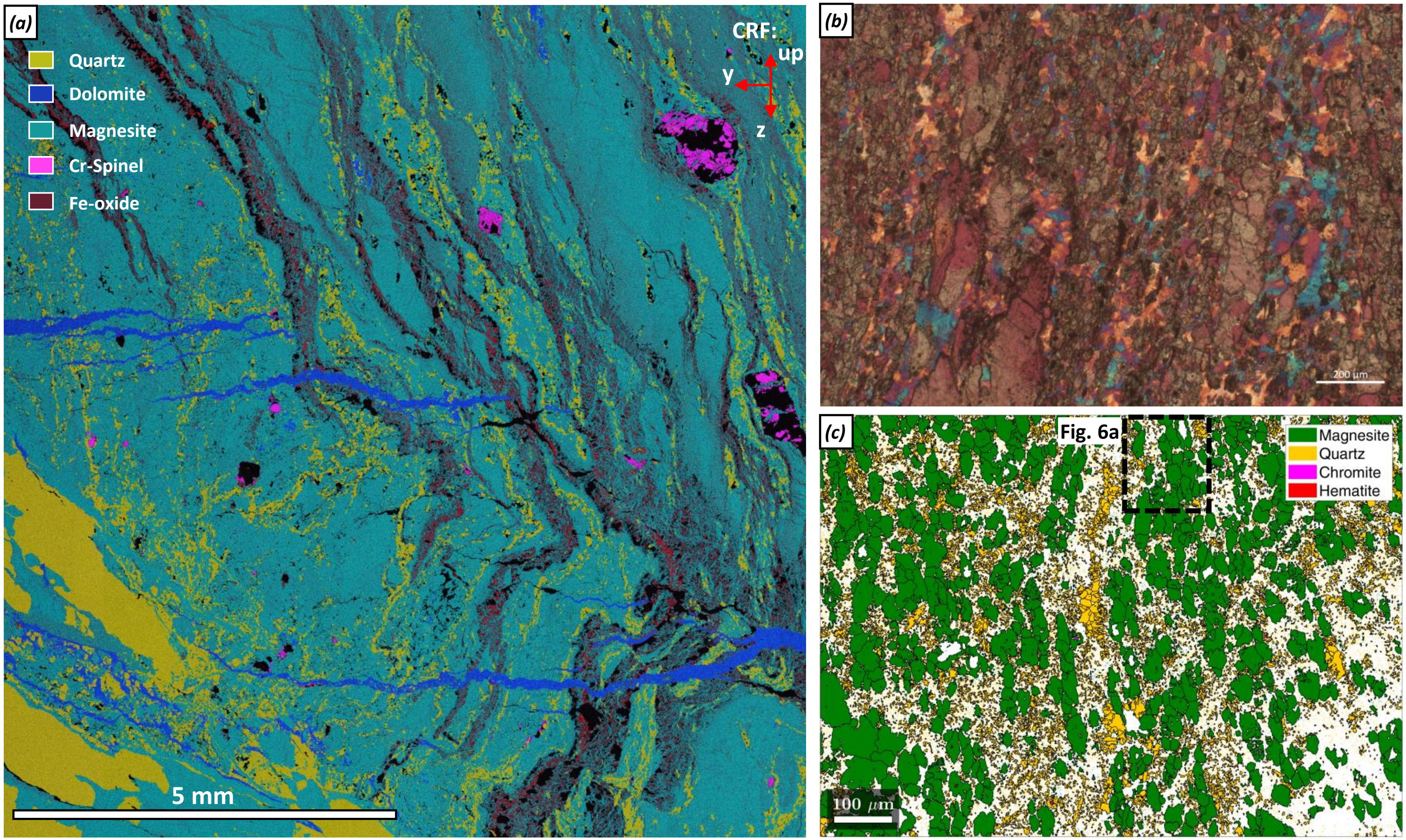

***Fig. S7** Foliated listvenite BT1B_14-3_60-66. (a) Composite-color EDS map of folded Fe-magnesite/hematite veins in foliated listvenite, with Cr-spinel porphyroclasts. Late dolomite veins cut the foliation; (b) aligned magnesite ellipsoids in quartz matrix (xpol with 1λ-plate); (c) EBSD phase map of an area where magnesite has an SPO, with the inset showing the location of Fig. 6a (see also Fig. S20).*

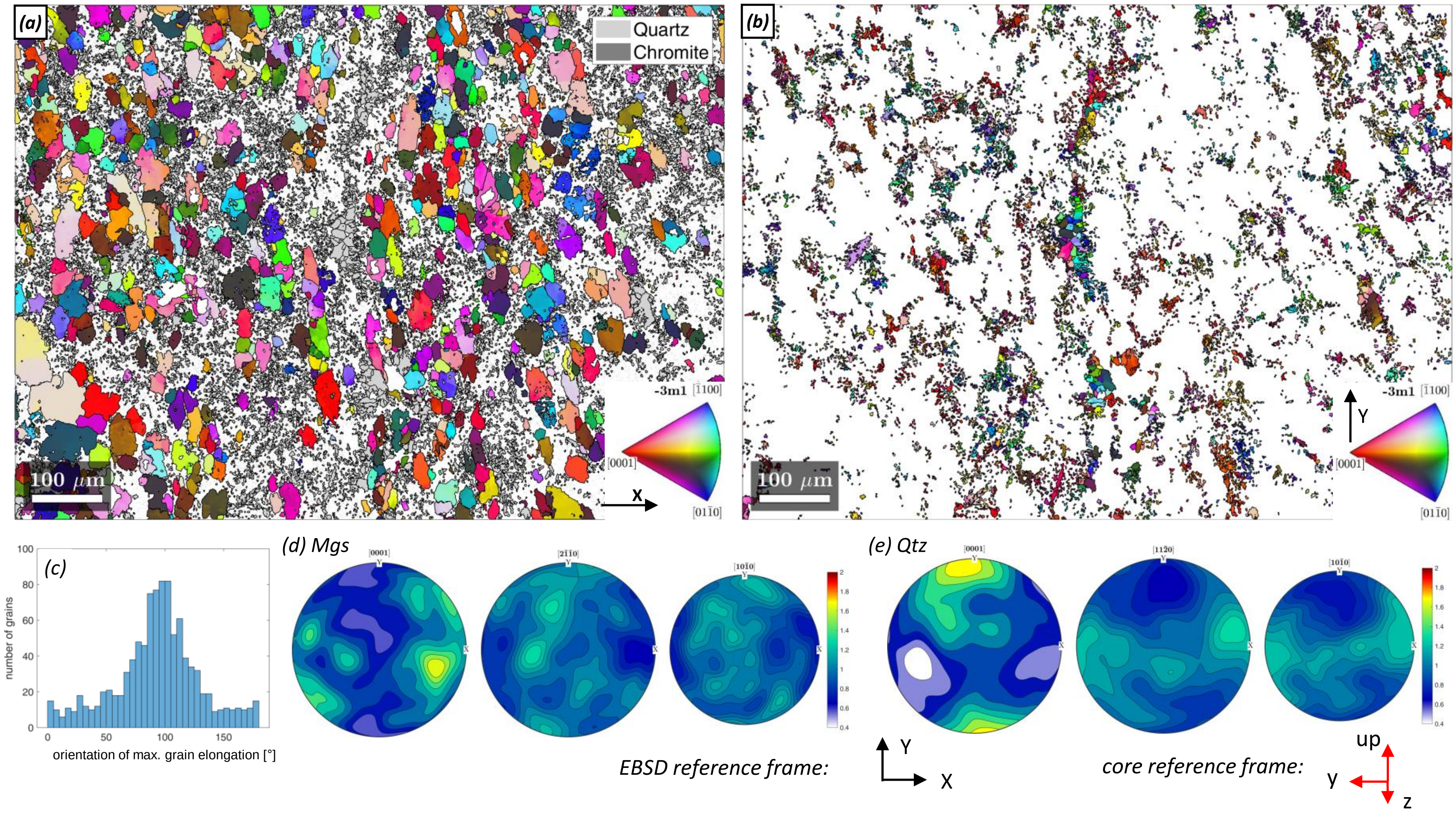

Fig. S8 *Shape and crystal preferred orientations in foliated listvenite (BT1B_14-3_60-66). (a) Magnesite orientation map (inverse polefigure colorscale see inset; showing each grain plotted in x direction). (b) Quartz orientation map (colorscale plotted in y direction). (c) Histogram of the grain elongation angle relative to the image horizontal in thin section, for magnesite with aspect ratio > 1.5. (e) Pole figures of the orientation distribution function for the c-, a- and m-axes of magnesite (n = 1351); polefigures of c-, a- and m-axes of quartz (n = 6754). Polefigures are lower hemisphere and one point per grain; the contour color scale is in multiples of a random distribution. The sample orientation is* Y_{EBSD} *= up-Hole (-z) in core reference frame (CRF), x(CRF) is into the plane.*

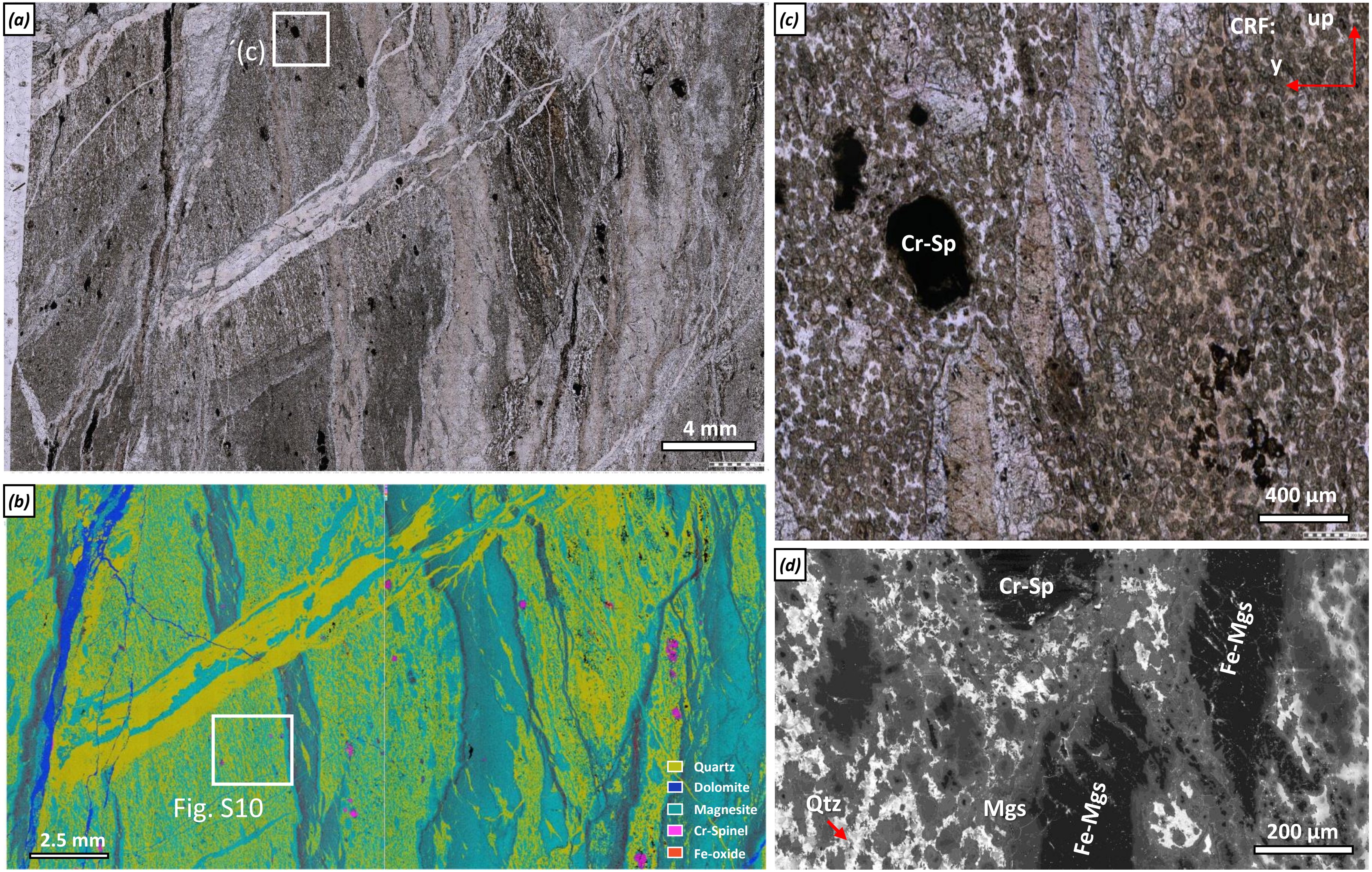

Fig. S9 *Foliated listvenite with transposed early magnesite veins (BT1B_14-3_77-80). (a) Thin section overview (ViP ppol); (b) composite-color EDS map showing early, locally transposed Fe-magnesite (Fe-Mgs) veins, cut by a quartz-magnesite and late dolomite vein. (c, d) detail of vein transposition, with consecutive magnesite generations overgrowing the transposed Fe-magnesite vein core (ViP ppol, and SEM-CL)*

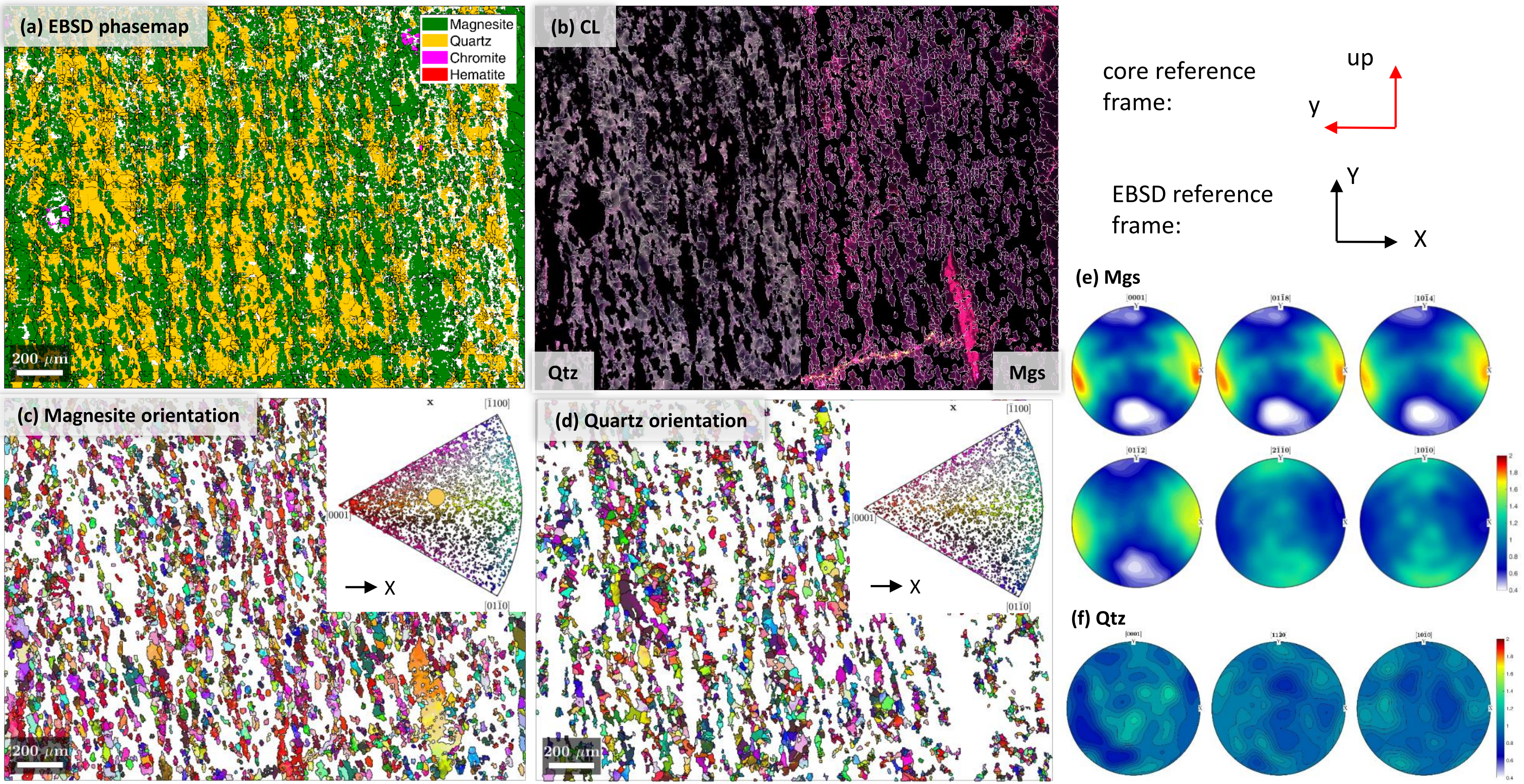

***Fig. S10** Shape and crystal preferred orientation of magnesite in foliated ellipsoidal listvenite (BT1B_14-3_77-80). (a) Aligned magnesite ellipsoids in quartz matrix; (b) optical CL image segmented for quartz (left) and magnesite (right), with grain boundaries from EBSD; magnesite has violet to pink luminescent, quartz shows two distinct luminescence colors. Single grains often contain both types of luminescence. (c) Magnesite orientation map, with colorscale based on the inverse polefigure (inset; showing each grain plotted in x direction and with point size relative to grain size); the relation to the core reference frame is given in the upper right; (d) Quartz orientation map and inverse polefigure; e) Pole figures of the orientation distribution function for the c-, e-, r-, f-, a- and m-axes of magnesite (n = 3205); polefigures of c-, a- and m-axes of quartz (n = 3060). Polefigures are lower hemisphere and one point per grain; the contour color scale is in multiples of a random distribution.*

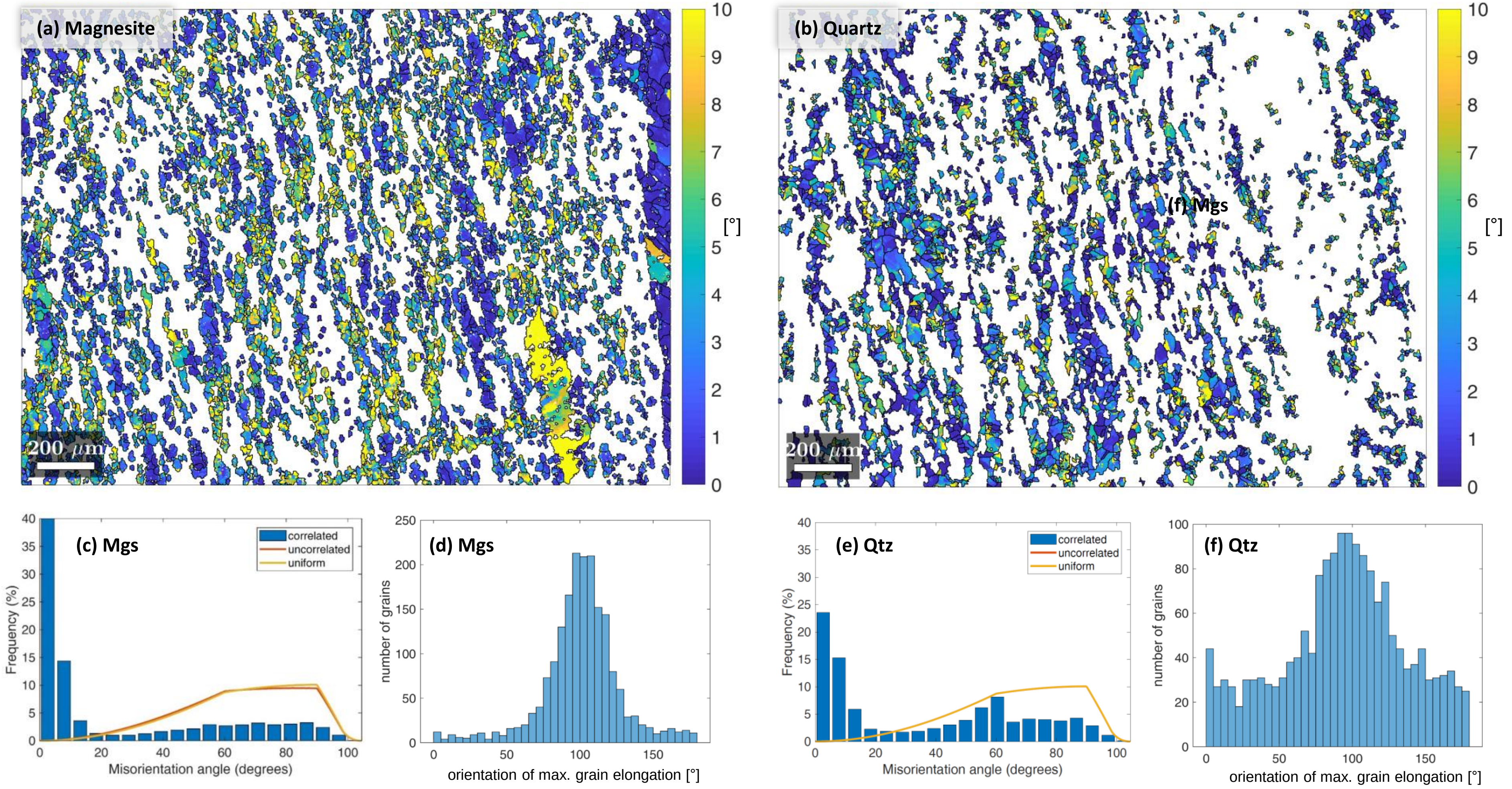

Fig. S11 *Misorientation to mean grain orientation maps for (a) magnesite and (b) quartz (BT1B_14-3_77-80; c.f. Fig. S10). (c, e) Misorientation-angle distribution between neighbouring (sub)grains (=correlated), related to every other grain (uncorrelated) and random (uniform) (grain segmentation angle 2°). (d, f) Histograms of the grain elongation angle relative to the image horizontal in thin section, for magnesite and quartz grains with aspect ratio > 1.5.*

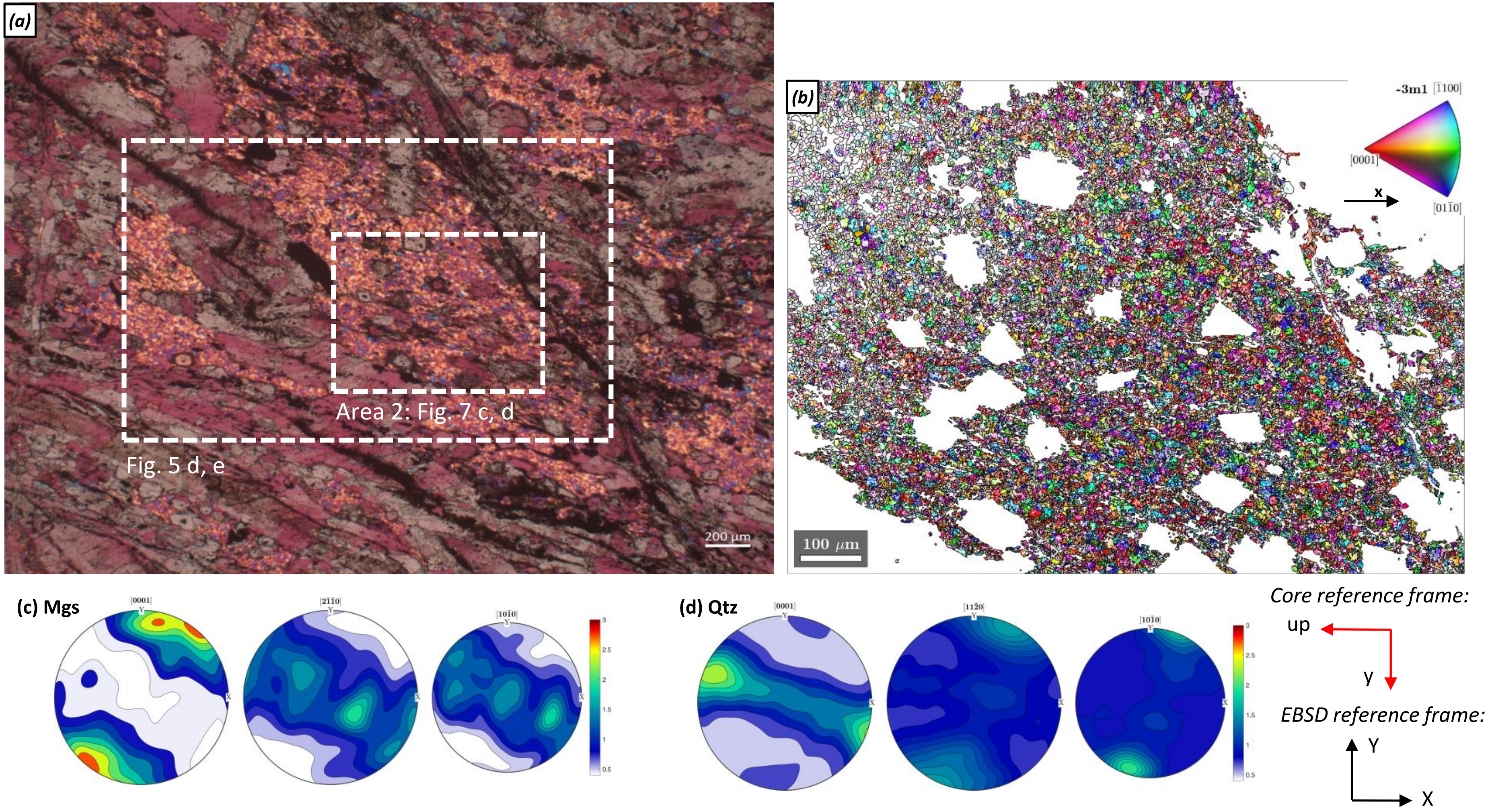

Fig. S12 *Folded early magnesite veins and fold axial cleavage in the matrix (BT1B_16-3_28-31). (a) crossed polarized micrograph with 1λ-plate; measured EBSD areas are marked; (b) Quartz orientation map and pole figures of quartz in EBSD area 2; (c) Polefigures of magnesite and (d) quartz in area 2, showing a CPO of quartz with [0001] parallel to the fold axial cleavage.*

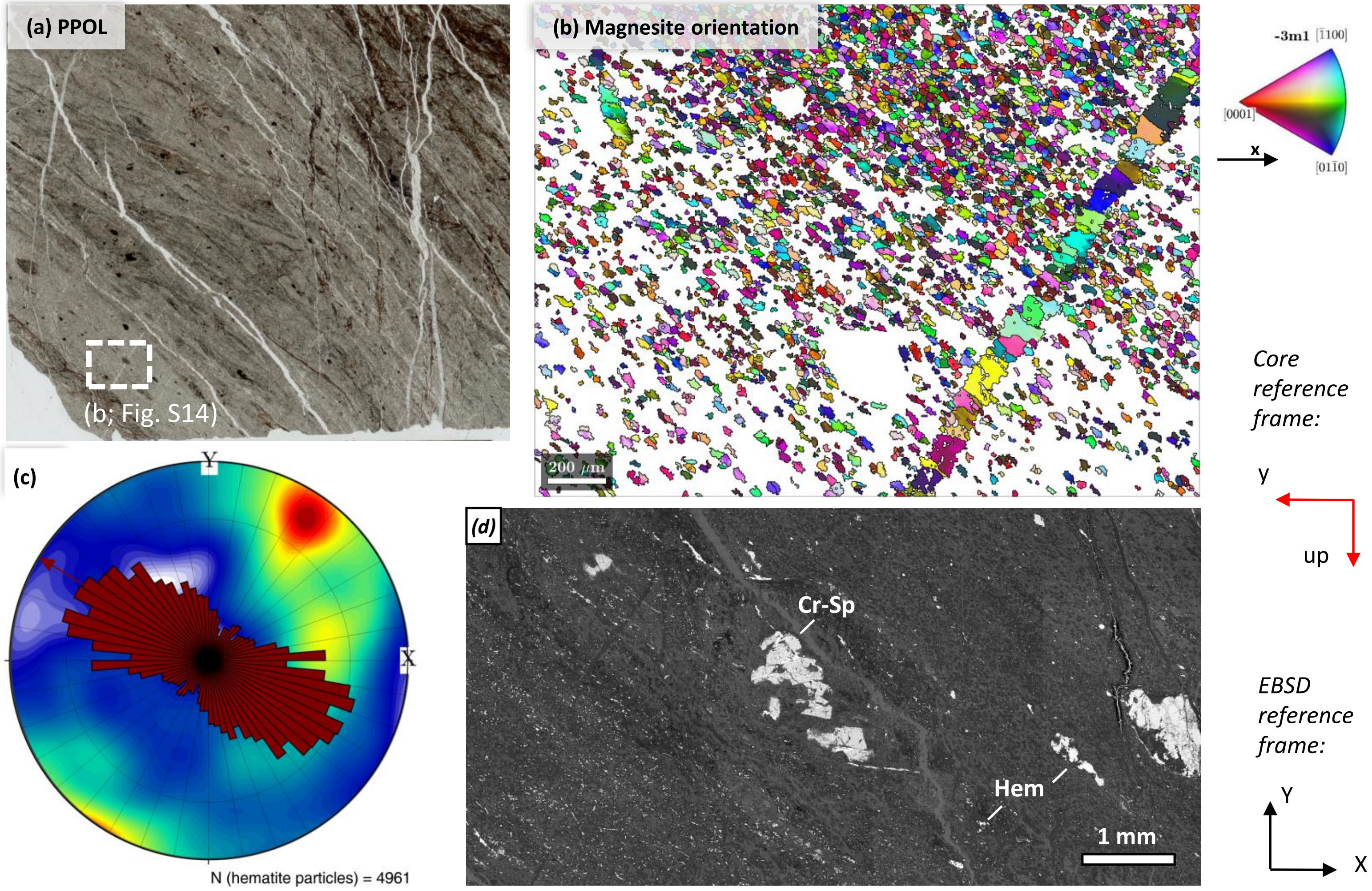

Fig. S13 *Shape and crystal preferred orientations in listvenite with mylonitic appearance (BT1B_20-1_64-68). (a) Thin section partial scan overview (image width 2.5 cm). (b) Magnesite orientation map colored according to the trigonal inverse pole figure color scale (see inset in upper right), in the area indicated in a). (c) Rose diagram of the orientation of the long axis of non-circular hematite grains in thin-section surface (from image analysis of a ViP reflected light scan), superposed on the magnesite c-axis pole figure from the EBSD map in b), excluding the vein (1 point per grain). (d) Large area BSE panorama, showing fragmented Cr-spinel in a foliated listvenite matrix with aligned hematite aggregates. Images and rose diagram are rotated into the EBSD reference frame for comparison.*

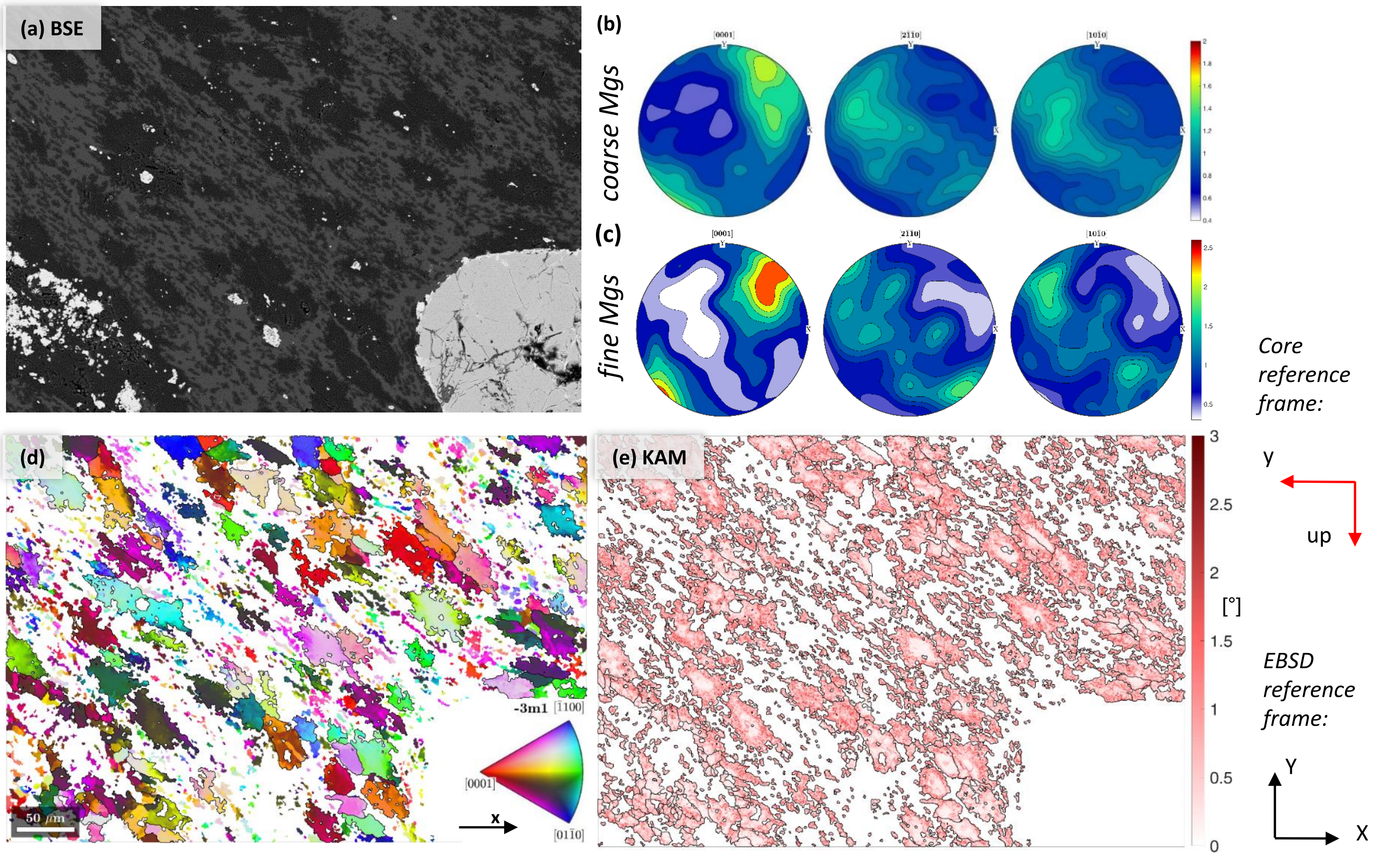

Fig. S14 *Shape and crystal preferred orientation of magnesite in listvenite with mylonitic appearance (small area in the marked field in Fig. S13a; sample BT1B_20-1_64-68). (a) BSE image showing bimodal grain size distribution of magnesite ellipsoids vs. aligned dendrites in the matrix. (b) Pole figure of coarse grained magnesite (n = 3254); (c) Pole figure of dendritic magnesite with EqR < 7 μm (n = 1338), (lower hemisphere, 1PPG). (d) magnesite orientation map colored according to the trigonal inverse pole figure color scale (x to E); fine grained magnesite is plotted without grain boundaries. (e) Kernel average misorientation thresholded to 3°.*

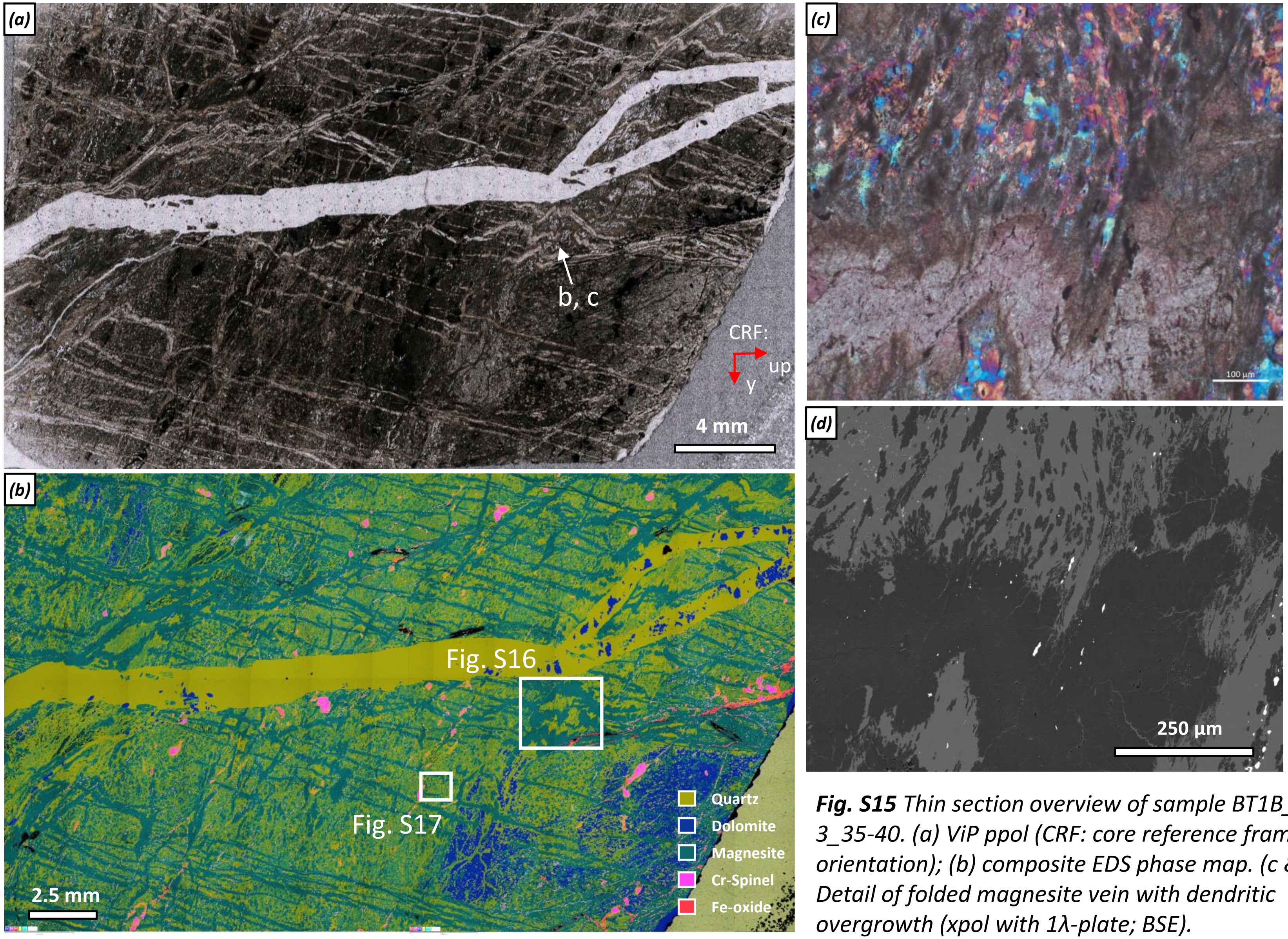

Fig. S15 *Thin section overview of sample BT1B_21-3_35-40. (a) ViP ppol (CRF: core reference frame orientation); (b) composite EDS phase map. (c & d) Detail of folded magnesite vein with dendritic overgrowth (xpol with 1λ-plate; BSE).*

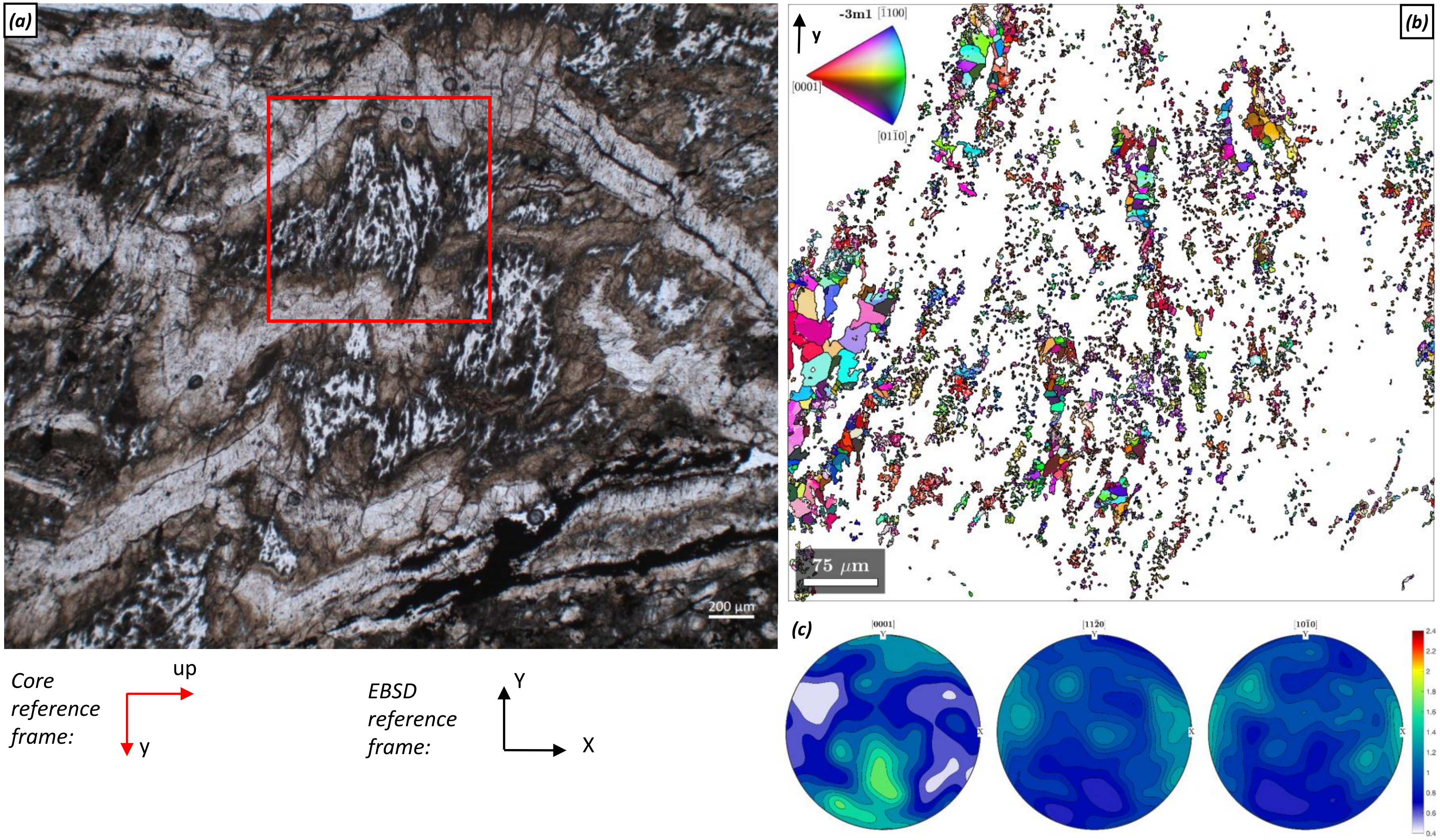

Fig. S16 *Folded early magnesite veins and fold axial cleavage in the matrix (BT1B_21-3_35-40). (a) plain polarized micrograph; (b, c) Quartz orientation map and pole figures of quartz (all points) in the area corresponding to the red frame, showing a weak local cpo of quartz with [0001] parallel to the fold axial cleavage.*

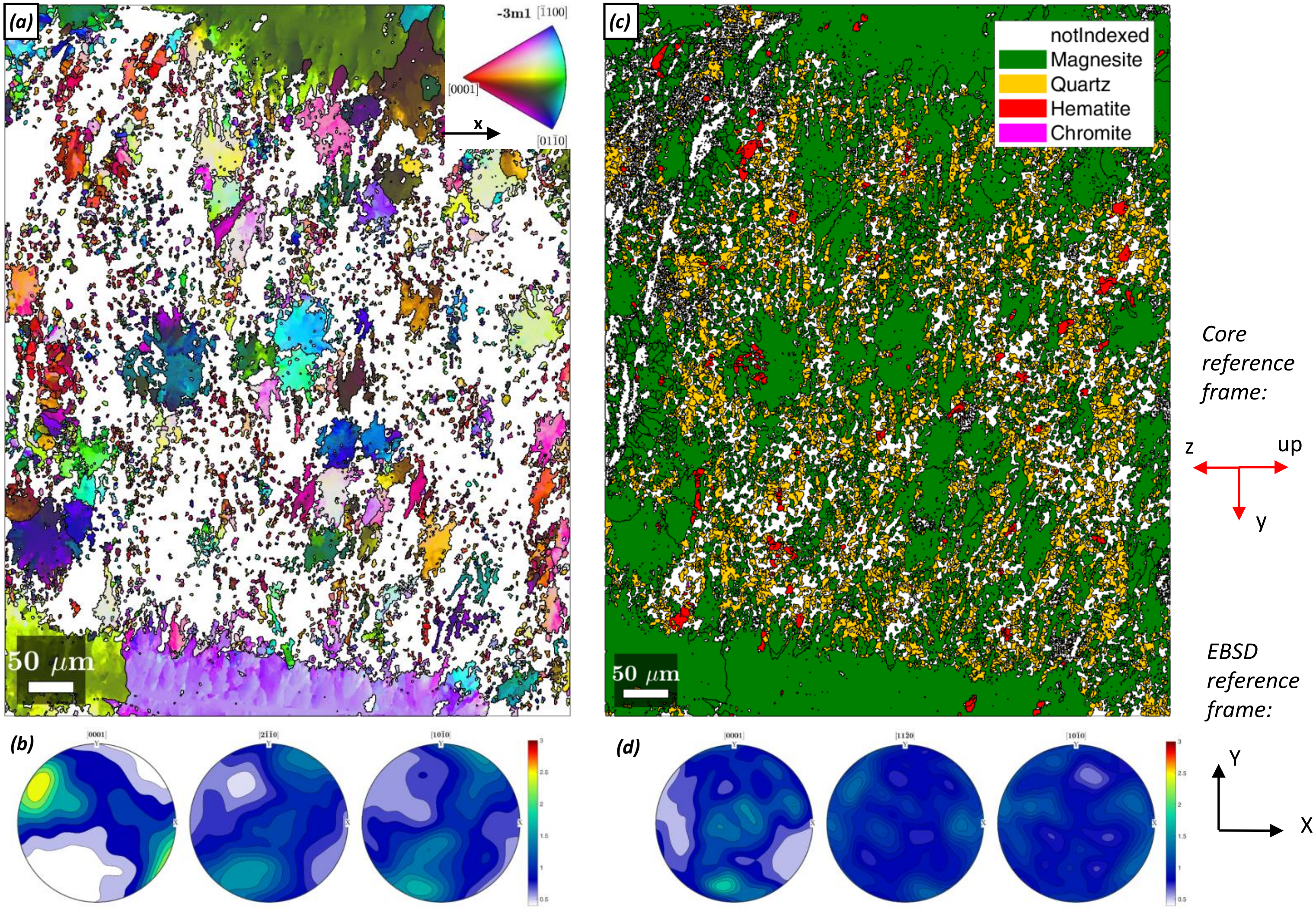

Fig. S17 *SPO and CPO in listvenite with strongly aligned magnesite dendrites (BT1B_21-3_35-40). (a) Magnesite orientation map colored according to the trigonal inverse pole figure color scale (inset, x to E); (b) Pole figures of magnesite (n = 3054); (c) EBSD phase map; (d) Pole figures of quartz (n = 2049). Pole figures are for the central part of the area only, excluding the magnesite veins at the top and bottom. Polefigures are lower hemisphere and one point per grain; the contour color scale is in multiples of a random distribution.*

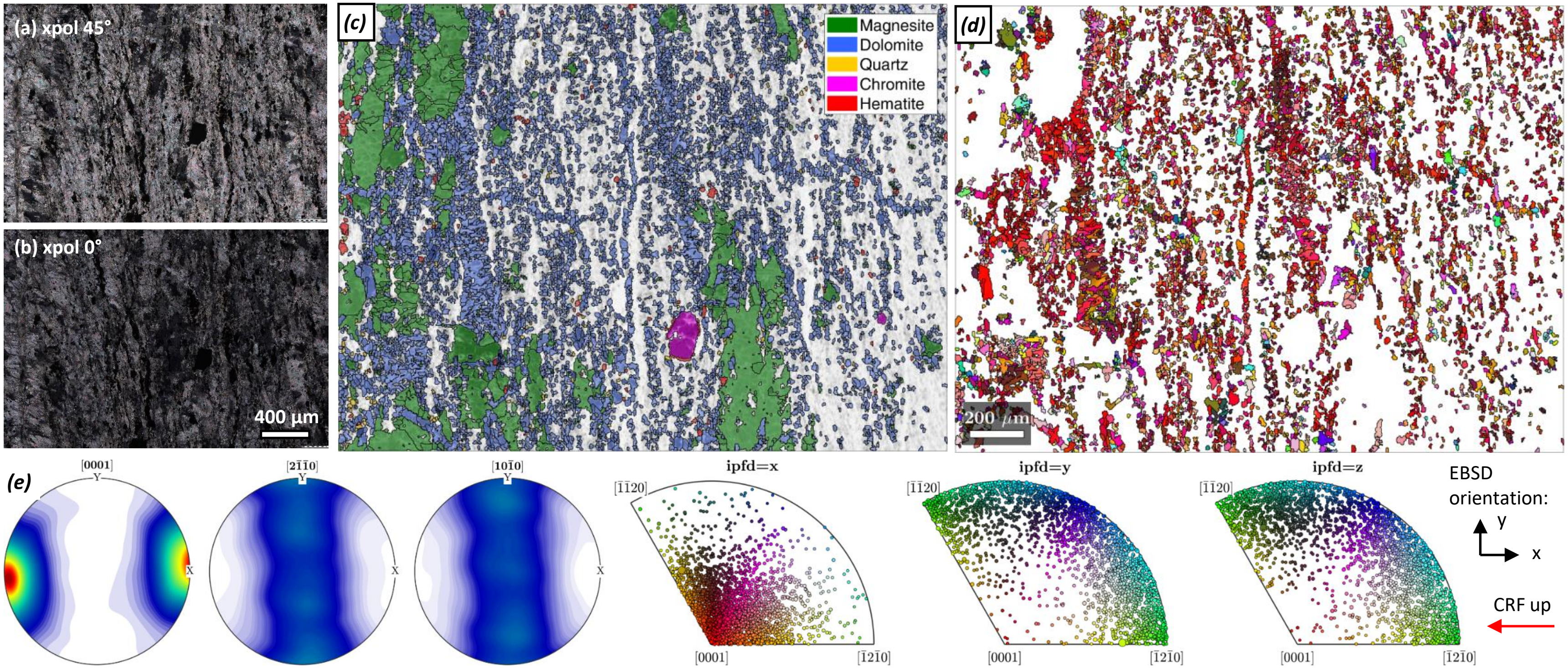

Fig. S18 *Strong CPO in foliated dolomite-rich listvenite close to the basal fault (BT1B_78-2_34-38). (a, b) ViP xpol images at 0 and 45° polarization angles, showing a cpo. (c) EBSD phase map; (d) dolomite orientation map (ipf colorscale to x; see inverse pole figures for colorscale); (e) 1-point-per-grain pole figure of orientation distribution function and inverse pole figures, showing a strong preferred orientation of dolomite with [0001] perpendicular to the foliation, and an a- / m- axes girdle distribution in the foliation plane. The EBSD x-axis corresponds here to the downdip direction of Hole BT1B (≈ 75° inclined).*

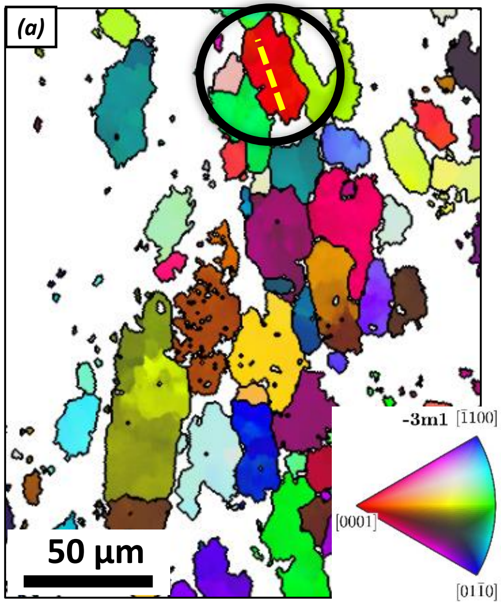

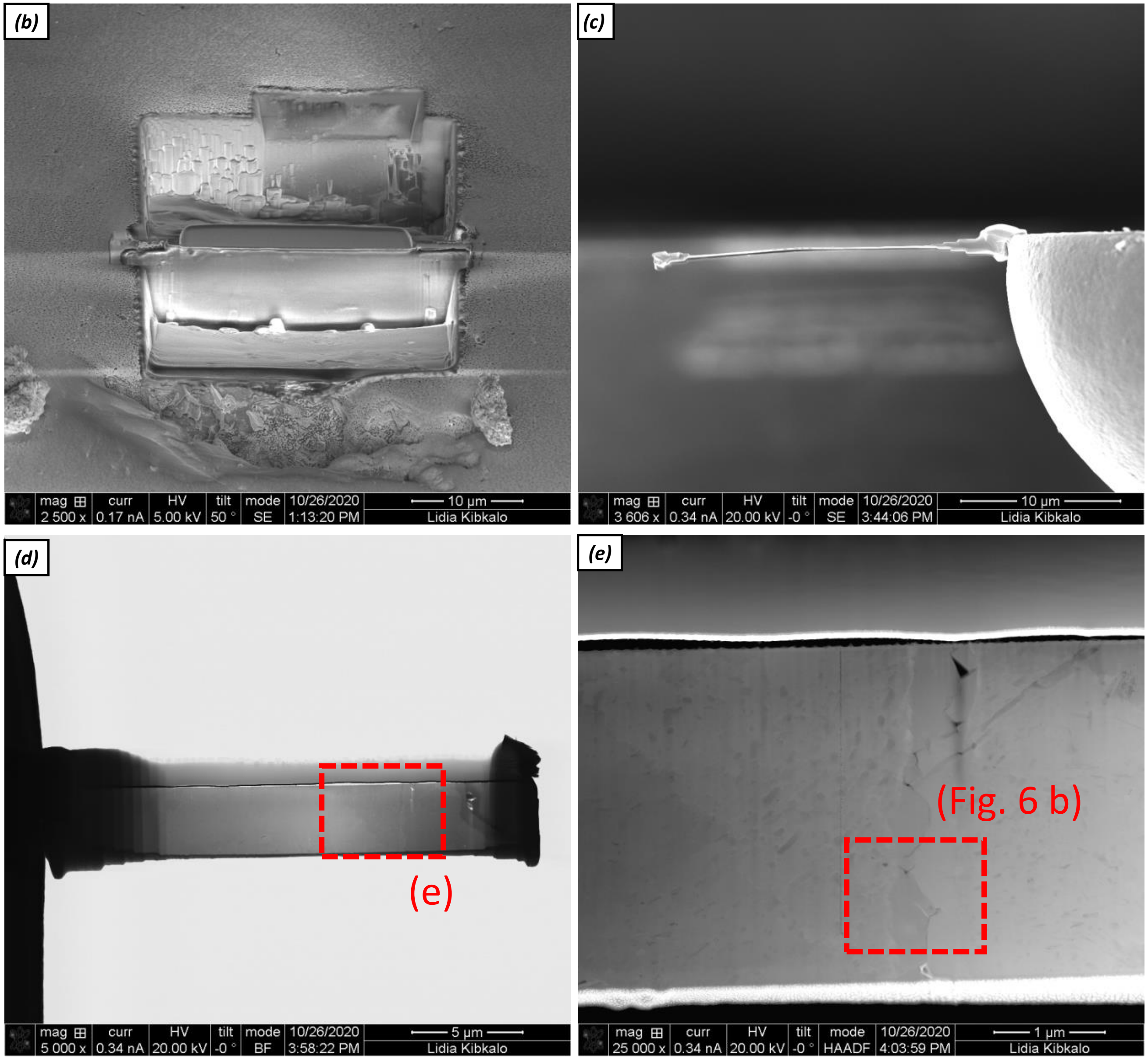

Fig. S19 *Location, FIB preparation and TEM images of ellipsoidal magnesite in foliated listvenite. (a) EBSD magnesite orientation map (orientation colorscale in z-direction with z out of plane); the FIB section was prepared parallel to the magnesite elongation direction and cutting several low-angle boundaries (black circle and yellow dotted line; c.f. Fig. 6 a). (b) FIP milling; (c) SEM image of FIB lamella attached to grid. (d) Bright-field image of the FIB section; (b) HAADF overview image of the area containing the sealed nano-fracture shown in Fig. 6 b. The sealed nano-fracture is oriented at high angle to the overall elongation direction of the magnesite grain.*

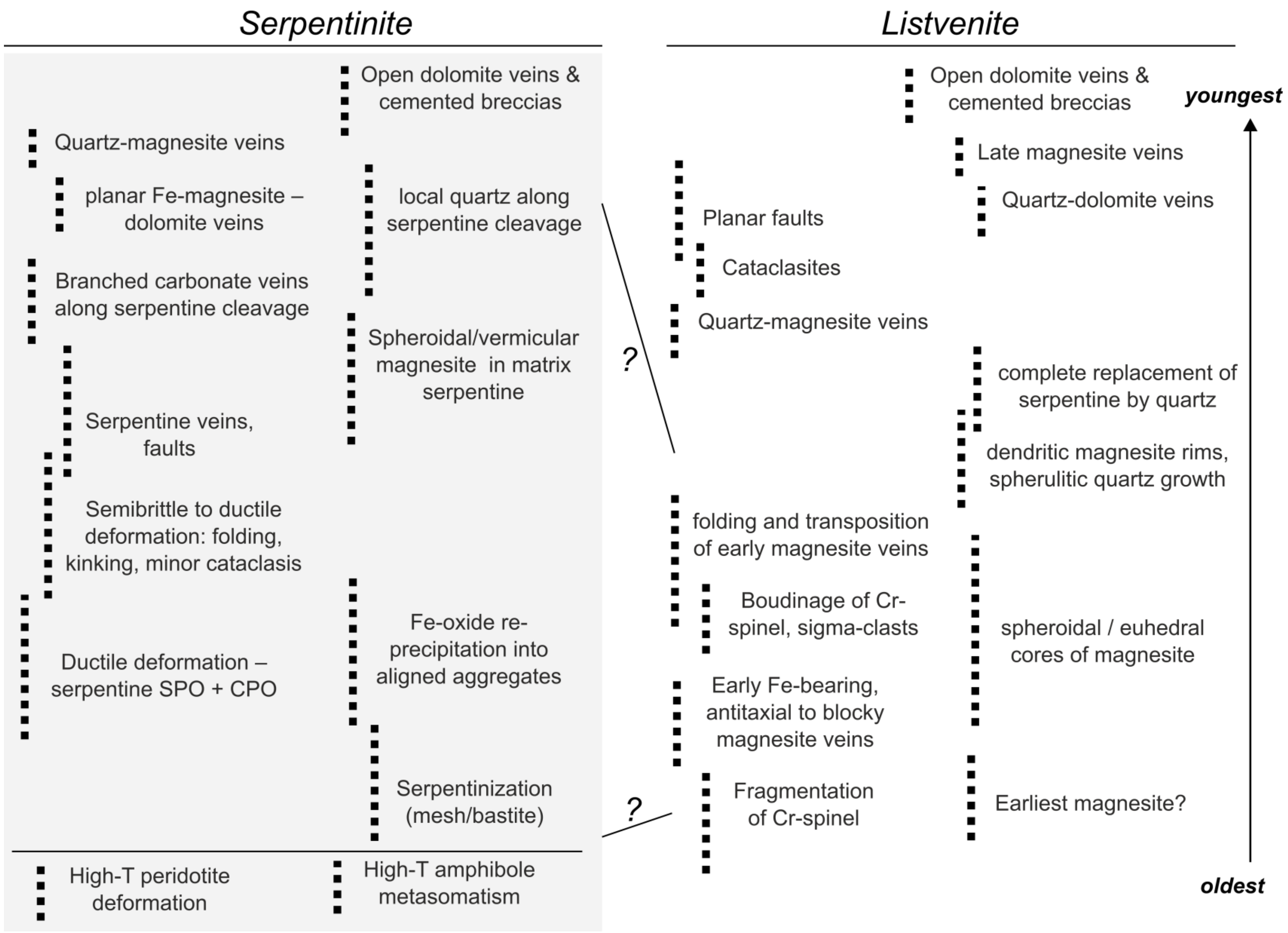

Fig. S20 *Relative age relations of carbonation reaction and deformation structures in serpentinites and listvenites of core BT1B. Cross-correlation between events in serpentinite relative to those in listvenite is uncertain.*
